# Stage‐Resolved Phosphoproteomic Landscape of Mouse Spermiogenesis Reveals Key Kinase Signaling in Sperm Morphogenesis

**DOI:** 10.1002/advs.202508538

**Published:** 2025-09-03

**Authors:** Tianyu Zhu, Yan Zhu, Xinyi Jiang, Xiangzheng Zhang, Bing Wang, Yu Chen, Yichen Zhao, Yulian Wang, Qi Zhou, Zhongyan Han, Yaling Qi, Mengjiao Luo, Haixia Tu, Bingjie Hao, Mengmeng Gao, Jie Ren, Xin Zhou, Xingyu Zhang, Xu Chen, Haojie Li, Qian Huang, Chenghao Situ, Yueshuai Guo, Hui Zhu, Yan Li, Xuejiang Guo

**Affiliations:** ^1^ State Key Laboratory of Reproductive Medicine and Offspring Health Department of Histology and Embryology Gusu School Nanjing Medical University Nanjing 211166 China; ^2^ Department of Bioinformatics Fujian Key Laboratory of Medical Bioinformatics School of Medical Technology and Engineering Fujian Medical University Fuzhou 350122 China; ^3^ Changzhou Maternal and Child Health Care Hospital Changzhou Medical Center Nanjing Medical University Changzhou Jiangsu 213000 China; ^4^ Department of Clinical Laboratory Sir Run Run Hospital Nanjing Medical University Nanjing 211166 China; ^5^ Innovation Center of Suzhou Nanjing Medical University Suzhou Jiangsu 215000 China

**Keywords:** CSNK1G1, kinase, phosphoproteomics, spermiogenesis, TTBK2

## Abstract

Spermiogenesis is the committed step of sperm production, during which spermatid cells undergo dramatic morphological transformations and transcriptional silencing. Post‐translational modifications (PTMs), including phosphorylation, provide a level of protein function flexibility and play important roles in spermiogenesis. Dynamic protein phosphorylation profiles of spermatids are characterized across four different developing steps, and identified phosphorylation regulation of key proteins in spermiogenesis. Expression module and kinase‐substrate phosphorylation network analysis revealed significant kinase activities of CSNK1G1 and TTBK2. CSNK1G1 is localized in the acrosome and is indispensable for acrosome biogenesis. *Ttbk2* male germ cell conditional knockout mice are infertile with flagella development and head shaping defects. TTBK2 is essential for both the phosphorylation and stabilization of IFT88, an intraflagellar transport (IFT) protein with which TTBK2 colocalizes and interacts. Ciliogenesis defects in *Ttbk2* knockout cells can be rescued by overexpression of TTBK2 or IFT88 but not kinase‐dead TTBK2. Collectively, the systematic profiling of the spermiogenesis phosphoproteome revealed the dynamic nature and important functions of kinase phosphorylation in spermiogenesis and male fertility.

## Graphical abstract

The quantitative proteomic and phosphoproteomic profiling reveals dynamic phosphorylation regulation of sperm morphogenesis. Kinase‐substrate phosphorylation network and phosphorylation module analysis, followed by in vivo knockdown and knockout analysis, identify TTBK2 and CSNK1G1 as key regulators of morphogenesis, including head, flagellar, and acrosomal development. This study provides insights into the temporal control of phosphorylation signaling during spermiogenesis, offering a valuable resource.

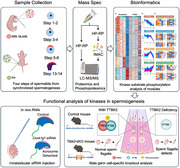

## 1 Introduction

About 17.5% of couples experience infertility, with male factors accounting for half of those cases.^[^ ^1^ ^]^ Oligo‐, astheno‐, teratozoospermia counts ≈25% of male infertility issues.^[^ ^2^ ^]^ Production of normal sperm depends on spermatogenesis, involving mitosis of spermatogonia, meiosis of spermatocytes, and spermiogenesis of spermatids.^[^ ^2^ ^]^ As the latest stage of spermatogenesis, mouse spermiogenesis can be divided into 16 well‐defined steps, and involves a series of drastic morphological changes and extreme chromatin condensation to develop from a round spermatid into mature sperm with tadpole‐like shape.^[^ ^3^ ^]^ Steps 1–8 encompass key processes such as chromatin condensation, acrosome biogenesis, and manchette assembly. Steps 9–16 correspond to the late phase of spermiogenesis, featuring manchette disassembly, elongation and condensation of the nucleus, and further flagellar development, to form mature sperm.^[^ ^4^ ^]^ Abnormalities of spermiogenesis can lead to oligo‐, astheno‐, teratozoospermia or even azoospermia. On account of dramatic molecular changes, including post‐meiotic sex chromatin repression^[^ ^5^ ^]^ and histone‐to‐protamine replacement,^[^ ^6^ ^]^ which happen in spermatids of this stage, gene transcription was gradually halted by nuclear condensation.^[^ ^7^ ^]^ Transcriptional silencing becomes pronounced at the late‐stage spermatids,^[^ ^8^ ^]^ and protein translation is paused, as mRNAs are stored in translationally repressed messenger ribonucleoproteins (mRNPs).^[^ ^7^ ^]^ Spermiogenesis is mainly regulated at the protein level, especially at the post‐translational modification (PTM) level. However, the present systematic studies have mainly focused on the transcriptional level; both protein level and post‐translational modification levels need further explication.

Functional analysis of individual proteins has shown the important roles of phosphorylation in spermiogenesis.^[^ ^9^ ^]^ Testis‐specific serine/threonine kinases (TSSKs) are crucial for spermiogenesis in mammals, playing a key role in the histone‐to‐protamine transition and affecting sperm nuclear shaping and flagellar organization by phosphorylating Protamine‐like protein MST77F/Ser9 and transition protein MST33A/Ser.^[^ ^10^ ^]^ Serine/threonine kinase 33 (STK33) phosphorylates fibrous sheath (FS) proteins A‐kinase anchoring proteins AKAP3/4 to assemble sperm flagella during spermiogenesis,^[^ ^11^ ^]^ making it a potential nonhormonal target for male contraception, as its inhibition leads to reversible defects in sperm morphology and motility without affecting testis size.^[^ ^12^ ^]^ HIPK4 is essential for male fertility as it regulates the phosphorylation of RIMBP3, a protein critical for sperm head shaping during spermiogenesis.^[^ ^13^ ^]^ However, systematic studies on the dynamics of protein expression and phosphorylation during spermiogenesis are lacking. It remains unknown whether the protein phosphorylation remains stable or is dynamically regulated during the different steps of spermiogenesis. The rapid advancement of mass spectrometry‐based proteomic techniques enables us to characterize the quantitative phosphoproteome from low amounts of samples in a high‐throughput manner.^[^ ^14^, ^15^ ^]^

Here, we profiled the dynamic phosphorylation landscape of purified spermatids synchronized at four different developing steps by using optimized sensitive quantitative phosphoproteomics techniques. In total, we quantified 3,681 differentially expressed proteins, along with 5,119 differentially regulated phosphorylation sites corresponding to 2,446 phosphorylation proteins. Proteins differentially regulated at the phosphorylation level are enriched in processes such as acrosome biogenesis, mRNA processing, sperm flagella, and chromatin remodeling. Kinase substrate phosphorylation network analysis revealed 27 kinases with substrate phosphorylation sites dynamically regulated. In vivo knockdown assay showed that CSNK1G1 is important for acrosome biogenesis and sperm progressive motility. Germ cell‐specific deletion of *Ttbk2* caused phosphorylation defect and instability of IFT88, leading to male infertility characterized by head shaping abnormalities and flagella defects in mice.

## 2 Results

### 2.1 Protein Abundance and Phosphorylation Landscapes of Spermatids Undergoing Spermiogenesis Across Four Developmental Steps

**Figure 1:**
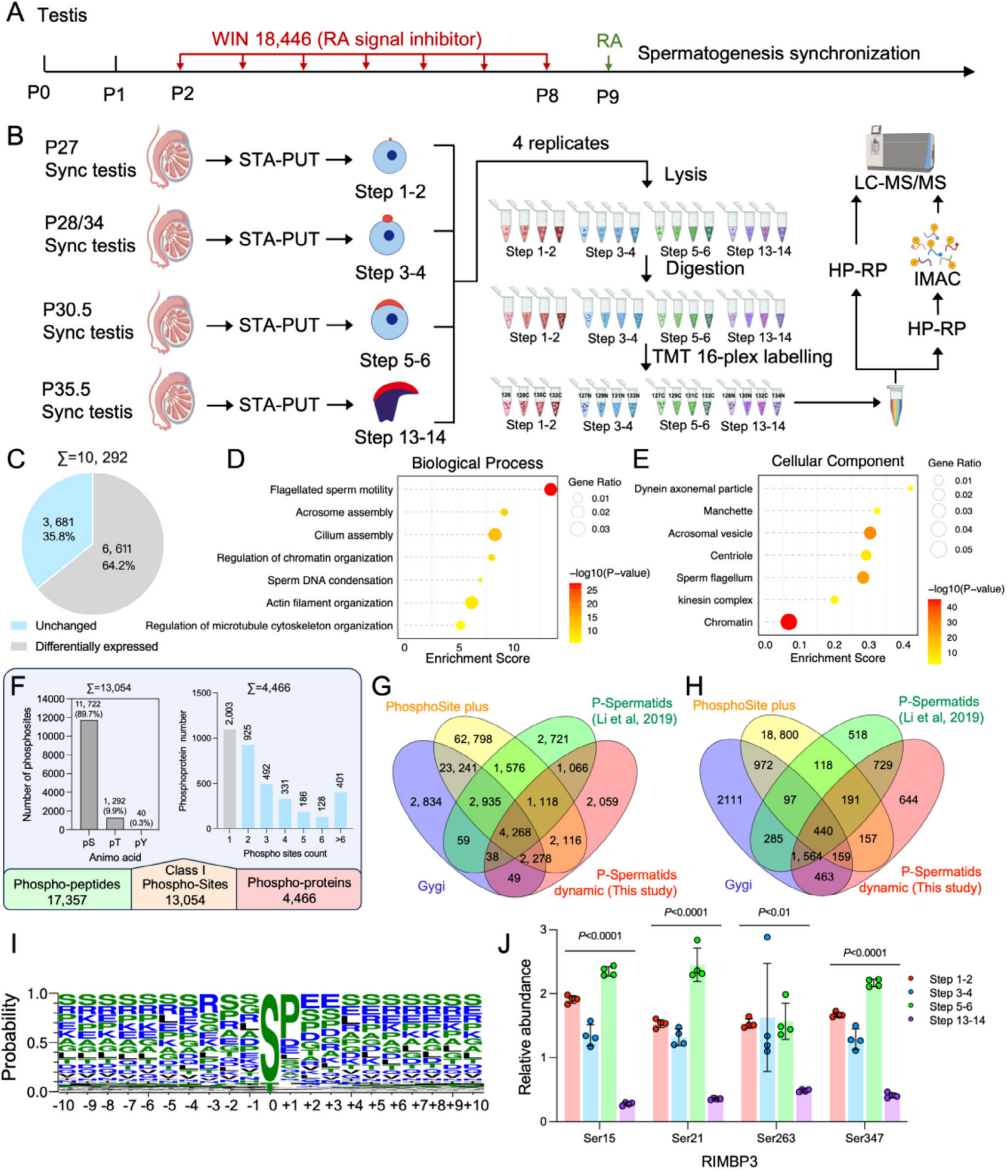
Quantitative phosphoproteomic profiling of mouse spermatid development A). Illustration of a strategy to obtain spermatogenesis synchronized testis. B). Workflow to profile quantitative proteome and phospho‐proteome of spermatids undergoing spermiogenesis across 4 different developing steps, each sample was labeled with a distinct TMT 16‐plex tag, allowing stage‐specific quantification in a single LC‐MS/MS run. The workflow diagram was created with BioRender.com, incorporating elements adapted from the template “qhps94u”, BioRender. Tianyu, Z. (2025) https://BioRender.com/qhps94u. C). Pie‐chart of proteins with differential abundance in the spermatids undergoing spermiogenesis. D‐E. Lollipop graph of enriched terms of biological process (D) and CC (E) in the differential proteins during spermiogenesis. F. Distribution of identified phosphorylated serine (pS), threonine (pT), and tyrosine (pY), number of phosphosites 21. This article is protected by copyright. All rights reserved. per protein, and numbers of phosphoproteins, phosphopeptides, and phosphosites in the spermatid phosphoproteome. G,H). The overlap of our phosphoproteome with Li et al.’s spermatid phosphoproteome (P‐Spermatids), Gygi's data, and PhosphoSitePlus database at site (G) and protein levels (H). For consistency and compliance of phosphopeptide data, we normalized the sequence window surrounding each phosphosite to 13 amino acids (± 6 residues). I). Sequence logo of 20 amino acids flanking identified phosphorylation sites. J). Phosphorylation dynamics of pSer15, pSer21, pSer263, and pSer347 on RIMBP3 during spermiogenesis. Relative abundance represents phosphorylation site level normalized to the corresponding protein level. Data are illustrated as mean ± SD (n = 4). Two‐tailed Student's t‐test was used for statistical analysis. The p‐values are labeled in the figure.

To obtain the homogeneous spermatids representing different developmental steps of spermiogenesis, we synchronized spermatogenesis in testis by N, N'‐1,8‐octanediylbis[N‐(4‐hydroxyphenyl) acetamide] (WIN 18,446) inhibition and retinoic acid (RA) initiation,^[^ ^16^, ^17^ ^]^ and purified spermatids at four different developmental steps using a gradient velocity sedimentation germ cell separation system called STA‐PUT (**Figure** **1**A,B). PNA staining of acrosomes and Hoechst staining of nuclei showed high purity (> 85%) of the isolated step 1–2 round spermatids (Step 1–2) with slight PNA positive proacrosomal vesicles (Figure S1A,B, Supporting Information), step 3–4 round spermatids (Step 3–4) with flattened acrosomes, step 5–6 round spermatids (Step 5–6) with cap‐like acrosomes, and step 13–14 elongated spermatids (Step 13–14) with elongated nuclei and dorsal fin‐like acrosomes.^[^ ^18^, ^19^ ^]^

Spermiogenesis is predominantly regulated at the protein level, including protein abundance and PTMs such as phosphorylation. To characterize the dynamic landscape of protein abundance and phosphorylation during spermiogenesis, we performed quantitative proteomic and phosphoproteomic analysis of the above isolated four steps of spermatids. Each sample was digested with trypsin and labeled with a distinct TMT 16‐plex isobaric tag, enabling stage‐specific quantification within a single LC–MS/MS run. The labeled samples were then pooled and subjected to high‐pH reversed‐phase fractionation, phosphopeptide enrichment, and LC–MS/MS analysis (Figure 1B). For both proteome and phosphoproteome, principal component analysis (PCA) showed high reproducibility among biological replicates within the same step, with step 13–14 samples clearly separated from earlier stages (Figures S2A and S3A, Supporting Information), indicating major molecular transitions during late spermiogenesis.

For the quantitative proteome, we identified 10,292 proteins, and found that 35.8% (3, 681/10,292) showed differential abundance [fold change (FC) > 2, FDR < 0.05] among the four steps of spermatids (Figure 1C; Table S1, Supporting Information). Those proteins with differential abundance were mainly enriched in sperm DNA condensation, acrosome assembly, regulation of chromatin organization, cilium assembly, centriole, sperm flagella, and manchette etc. (Figure 1D,E; Table S2a,b, Supporting Information). We also identified 150 proteins with differential abundance between step 1–2 and step 3–4, 313 between step 3–4 and step 5–6, and a marked increase to 2,701 between step 13–14 and step 5–6 (Table S1 and Figure S2B, Supporting Information). We performed gene ontology (GO) enrichment analysis on proteins with differential abundance between adjacent stages. From step 1–2 to 3–4, altered proteins were enriched in sperm motility, spermatid differentiation, fertilization, acrosomal vesicle, nucleosome, and microtubule‐related processes (Figure S2C,D, and Table S2b, Supporting Information). Between steps 3–4 and 5–6, proteins related to chromatin regulation, flagellar structures, CatSper complex, acrosome biogenesis, and cytoplasmic microtubules were enriched (Figure S2E,F, and Table S2c, Supporting Information). From step 5–6 to 13–14, proteins involved in RNA splicing, heterochromatin formation, nuclear organization, cilium assembly, and fertilization became increasingly prominent (Figure S2G,H, and Table S2d, Supporting Information).

For the quantitative phosphoproteome, we quantified 13,054 phosphorylation sites (Figure 1F) corresponding to 4,466 phosphorylated proteins in four steps of spermatids (Figure 1F; Table S3a, Supporting Information). Compared with previously published Li et al.’s spermatid phosphoproteome,^[^ ^9^ ^]^ Gygi et. al.’s phosphoproteome of multiple mouse tissues,^[^ ^20^ ^]^ and PhosphoSitePlus database,^[^ ^21^ ^]^ 2,059 phosphorylation sites were newly identified in our study, and 644 proteins were newly identified to be phosphorylated (Figure 1G,H). To reveal the feature of the phosphorylation sites identified in this study, we analyzed amino acids distribution. The results showed that the percentages of phosphoserine (pS), phosphothreonine (pT), and phosphotyrosine (pY) were 89.7%, 9.9%, and 0.3%, respectively (Figure 1F). And 55% (2,465 out of 4,466) of the phosphorylated proteins possessed two or more phosphosites. Notably, ≈9% (401 out of 4,466) of these proteins exhibited more than six phosphosites, suggesting that multi‐phosphorylation is prevalent during spermatid development (Figure 1F). We further analyzed the sequence feature surrounding phosphorylation sites using WebLogo,^[^ ^22^ ^]^ and found a dominant phosphorylation motif of proline following phosphoserine or phosphothreonine (Figure 1I).

Previous studies have reported that Ser15, Ser21, Ser263, and Ser347 of RIMBP3 are highly phosphorylated during spermiogenesis and play a critical role in manchette formation.^[^ ^13^ ^]^ Our phosphoproteomic analysis revealed elevated phosphorylation levels at steps preceding manchette formation (step 1–2, step 3–4, and step 5–6), compared to steps following manchette disassembly (step 13–14). These observations supported the findings of previous studies and further affirmed the reliability of our dataset (Figure 1J). Here, with the dynamic phosphoproteomics data as a foundation to explore novel phosphorylation characteristics, we aim to elucidate the potential implications of phosphorylation dynamics in spermatid development.

### 2.2 Dynamics of Differentially Regulated Protein Phosphorylation During Spermatid Development

**Figure 2:**
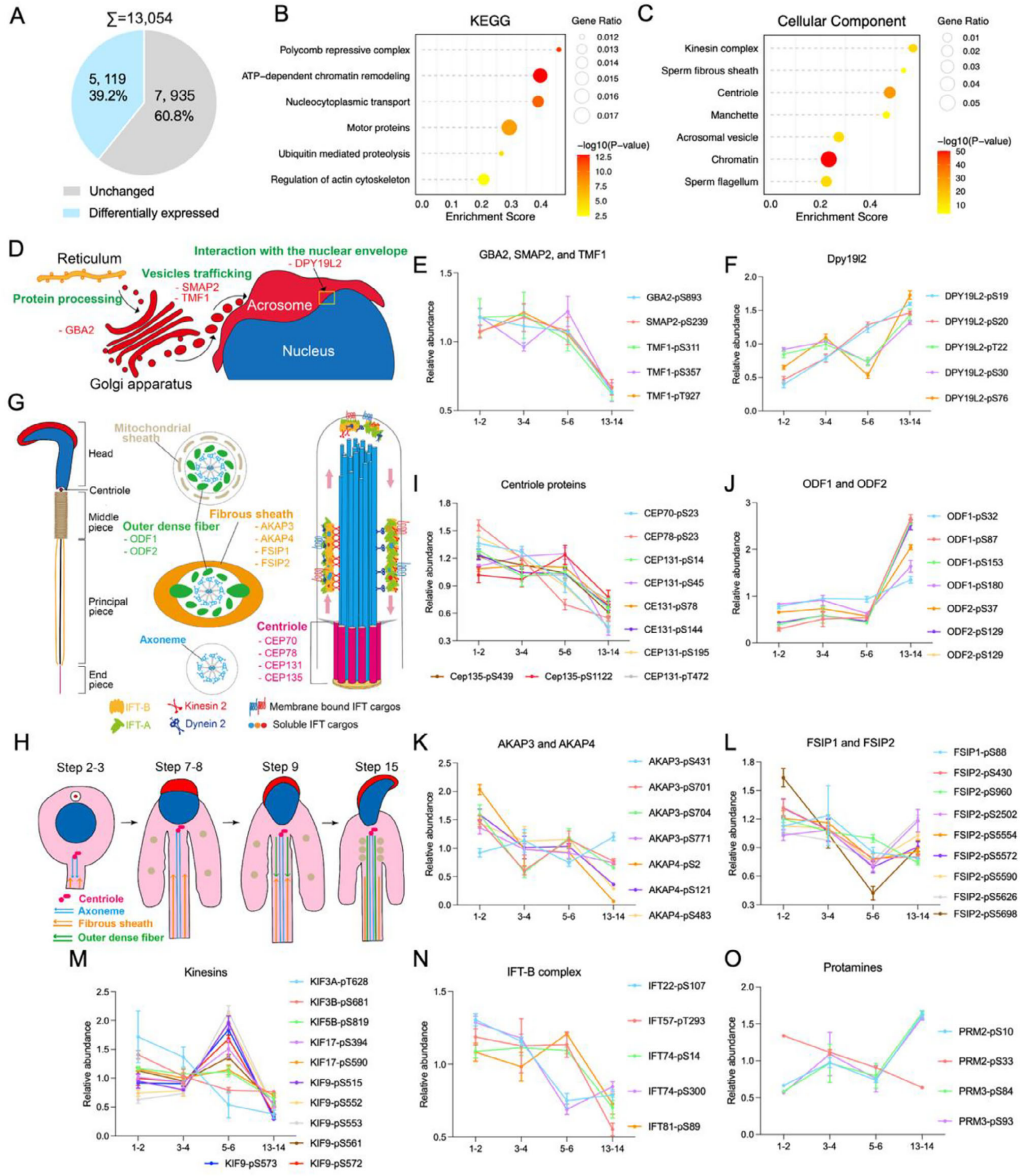
Enrichment and annotation analysis of the differential phosphoproteome of spermiogenesis. A). Pie‐chart of proteins with differentially regulated phosphorylation sites in the spermatids undergoing spermiogenesis. B‐C. Lollipop graphs of the statistically enriched KEGG pathways (B) and CC (C) in the differential phosphoproteome. D). Schematic representation of acrosome biogenesis. E,F). Relative abundance (mean ± SD based on 4 biological replicates) changes of phosphorylation sites in acrosomal proteins across developmental stages. G,H). Schematic models of the flagellar axoneme (G) and peripheral structure formation (H) during spermiogenesis. I–O). Relative abundance (mean ± SD based on 4 biological replicates) changes of phosphorylation sites in centriolar proteins (I), outer dense fiber proteins ODF1 and ODF2 (J), FS proteins (K‐L), kinesins (M), IFT complex B (IFT‐B) proteins (N), and protamines (O) across four developmental stages of spermatids. Phosphosite positions (e.g., pS357) refer to the position of phosphorylated serine residues of the corresponding protein. Relative abundance of phosphosites was calculated as the phosphosite level normalized to the protein level of the corresponding protein.

Above omics profiling suggested that spermiogenesis involves complex phosphorylation regulation, to further evaluate the phosphorylation dynamic changes during spermiogenesis, we performed differential analysis of phosphorylation sites and identified 5,119 phosphorylation sites differentially regulated among the four stages of spermatids [fold change (max/min) > 2, FDR < 0.05] (**Figure** **2**A; Table S3, Supporting Information). These phosphoproteins are predominantly enriched in cellular component terms related to chromatin, nucleocytoplasmic transport, ATP‐dependent chromatin remodeling, motor proteins, ubiquitin‐mediated proteolysis according to KEGG pathway analysis, and are enriched in localizations of sperm flagella, sperm FS, centriole, acrosomal vesicle, and kinesin complex, etc. (Figure 2B,C; Table S4a, Supporting Information). We also identified 164 differentially regulated phosphorylation sites between step 1–2 and step 3–4; 307 differentially regulated phosphorylation sites between step 3–4 and 5–6; 3,654 differentially regulated phosphorylation sites between step 5–6 and 13–14 (Table S3 and Figure S3B, Supporting Information). GO enrichment analysis of differentially regulated phosphosites in adjacent steps revealed enrichment in sperm DNA condensation, flagellar motility, fertilization, actin cytoskeleton, nucleosome, and ciliary structures between step 1–2 and 3–4 (Figure S3C,D and Table S4b, Supporting Information), in acrosomal vesicle, cilium assembly, nucleosome organization, kinesin complex, and RNA splicing showed dynamic regulation between step 3–4 and 5–6 (Figure S3E,F and Table S4c, Supporting Information), and in RNA processing, heterochromatin, centromeric chromatin, and nuclear chromosome structure between step 5–6 and 13–14 (Figure S3G,H and Table S4d, Supporting Information). Together, quantitative proteomic and phosphoproteomic profiling reveal enrichment in both common and stage‐specific processes during spermatid development. Flagellum and acrosome assembly span multiple stages, while late spermiogenesis is marked by RNA processing and chromatin remodeling. Surprisingly, fertilization‐related pathways are enriched even at early stages, suggesting early molecular preparation for fertilization.

Proteins involved in different events of spermiogenesis may have different quantitative phosphorylation patterns. We analyzed proteins with well‐known functions in key biological events, including the biogenesis of acrosome and flagella, and protamine substitution of histone. The development of the acrosome occurs throughout the whole process of spermiogenesis (Figure 2D). Our findings indicate that the phosphorylation pattern of certain proteins is more prominently associated with specific steps of acrosome development. Proteins such as GBA2, SMAP2, and TMF1 exhibit a decrease in phosphorylation during spermiogenesis, consistent with their roles in the initial assembly of acrosomal components (Figure 2E).^[^ ^23^ ^]^ Conversely, DPY19L2, a protein crucial for mediating the interaction between the acrosome and the nuclear envelope, displays elevated phosphorylation at step 13–14 of spermatid development, correlating with the later steps of acrosomal maturation and integration (Figure 2F).^[^ ^23^ ^]^

The sperm flagella are the key structure that ensures the motility of sperm, its biogenesis initiates at early steps, and elongation of the flagella axoneme starts at the centriole (Figure 2G).^[^ ^24^ ^]^ Our dataset revealed a higher phosphorylation level of centriolar proteins CEP70, CEP78, CEP131, and CEP135 at the early step of spermiogenesis (Figure 2I). Apart from the axoneme, outer dense fiber (ODF) and FS are also the unique structures in sperm flagella, and are incorporated during axoneme extension, with FS formation from step 2,^[^ ^25^ ^]^ and ODF assembly from step 9^[^ ^4^ ^]^ (Figure 2J). In our dataset, the phosphorylation level of the ODF protein progressively increased as spermatids developed, peaking at step 13–14 (Figure 2J). Conversely, the phosphorylation level of both FS assembly essential kinase AKAP3/4 (Figure 2K) and the FS component FSIP1/2 (Figure 2L) gradually decreased as spermiogenesis proceeded, suggesting that phosphorylation regulates the assembly of these two structures.

**Figure 3:**
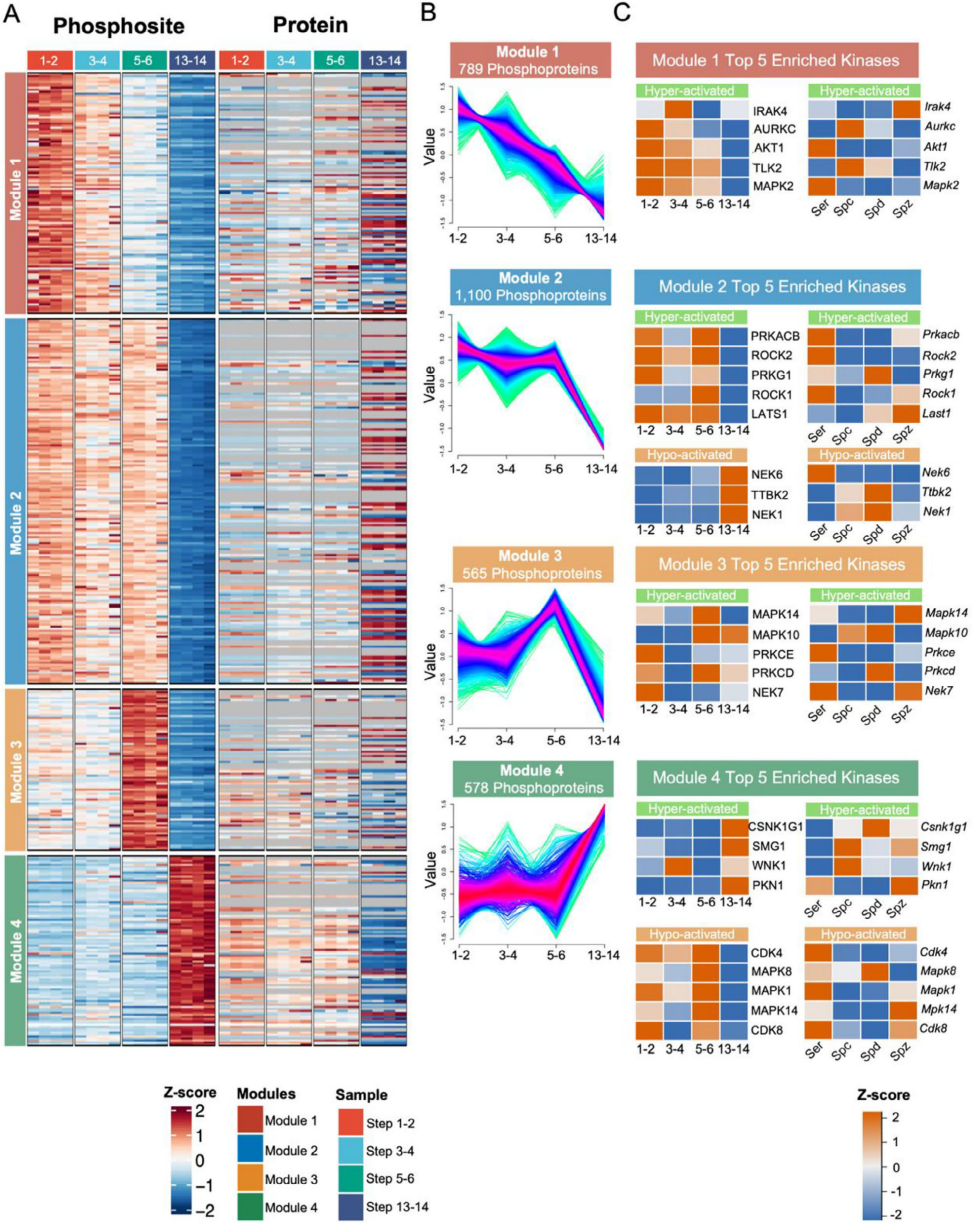
Cluster and Kinase substrate phosphorylation network enrichment analysis of distinct phosphorylation modules among different steps of spermatids. A). Unbiased hierarchical cluster analysis of differentially regulated phosphoproteome demonstrates distinct protein phosphorylation and expression profiles during spermatid development (steps 1–2, 3–4, 5–6 and 13 14). B). Profile plot of four phosphorylation pattern clusters during spermatid development. C). Heatmap of protein abundance (left) and mRNA expression (right) levels of kinases with significantly hyporepresented or hyperrepresented substrate phosphorylation sites in each module. Ser, sertoli; Spc, spermatocyte; Spd, spermatid; Spz, spermatozoa.

The structural components of the sperm flagella are transported and assembled via a bi‐directional movement IFT mechanism (Figure 2H). Interestingly, the protein phosphorylation pattern varies with different transport directions. Phosphorylation levels of most phosphorylation sites of anterograde transporting Kinesin and IFT‐B decreased during spermiogenesis (Figure 2M,N), while the phosphorylation level of KIF9 and KIF17 exhibited an elevation at step 5–6 (Figure 2M). In contrast, the phosphorylation patterns of retrograde transporting Dynein and IFT‐A are more diverse (Figure S3I,J, Supporting Information), the phosphorylation levels of IFT140 and TTC21A gradually increased, while the phosphorylation level of IFT122 increased significantly at step 5–6 (Figure 3I, Supporting Information). Regarding myosin, another motor protein, most myosin‐related phosphosites decreased at step 13–14, whereas MYO1C‐pS408 increased despite stable MYO1C protein levels across steps. This suggests a stage‐specific regulatory role of MYO1C‐pS408 in late spermiogenesis (Figure 3K, Supporting Information).

Dramatic changes also take place in the spermatid head, where histones are replaced by protamines. The dynamic change in protamine phosphorylation levels primarily occurs in PRM1, with its phosphorylation levels increasing significantly during step 13–14 (Figure 2O). Collectively, phosphorylation of key proteins in spermiogenesis is highly dynamic during spermatid development, providing a crucial perspective of developmental control at the post‐translational level.

### 2.3 Kinase‐substrate Phosphorylation Network Analysis Revealed Module‐specific Kinases in Spermiogenesis

Spermiogenesis is controlled through precisely timed and highly organized cycles. To further explore the temporal regulation of phosphorylation, we clustered stage‐specific phosphosites using Mfuzz. The phosphorylation sites can be clustered into four modules, with their protein abundance was not in a similar trend (**Figure** **3**A,B; Tables S1 and S3, Supporting Information), indicating distinct regulation at the phosphorylation level from the expression level. Protein phosphorylation can be gradually down‐regulated (module 1), down‐regulated only at step 5–6 (module 2), peaked at step 5–6 (module 3), or peaked at step 13–14 (module 4) (Figure 3A; Tables S1 and S3, Supporting Information). In the four modules, module 2 and module 4 have completely opposite trends, indicating rich protein regulation dynamism and diversity in spermiogenesis.

Protein phosphorylation is catalyzed by a kinase. To identify important regulatory kinases for different phosphorylation dynamic patterns, the cluster‐specific phosphorylation sites were subjected to GPS^[^ ^26^ ^]^ for kinase substrate annotation and substrate enrichment analysis. The results showed that the kinases with hyperrespresented substrates are supposed to have high kinase activity in the corresponding module, and are supposed to have a similar expression pattern to the substrates. In total, phosphorylation clustering followed by kinase substrate phosphorylation network analysis revealed 27 significant kinases with substrate phosphorylation sites dynamically regulated in 4 different modules. While the kinases with hyporepresented substrates are supposed to have low kinase activity. Among the significant kinases, we systematically prioritized kinase candidates with putative regulatory roles in spermiogenesis by integrating phosphoproteomic dynamics, protein abundance trends from our phophoproteomics data and proteomics data, and testicular cell‐type specific transcriptomic data from a published database.^[^ ^27^ ^]^ We further analyzed the expression pattern of these kinases, and found that kinases having hyperrespresented substrates tend to have an expression pattern similar to the substrates in the module (Figure 3C; Table S5, Supporting Information). While the kinases having hyporepresented substrates tend to have an expression pattern opposite to the module, with their protein abundance pattern in 4 different steps, spermatids. To further ensure biological relevance and cell‐type specificity, we cross‐referenced spermatogenic cell‐type resolved RNA‐seq data^[^ ^27^ ^]^ and selected kinases with enriched expression in spermatids rather than spermatocytes and Sertoli cells(Figure 3C). This integrative approach yielded five kinases: protein kinase cGMP‐dependent 1 (PRKG1) and large tumor suppressor kinase 1 (LATS1) in Module 2, Tau tubulin kinase 2 (TTBK2) in Module 2, Protein kinase C‐delta (PRKCD) in Module 3, and Casein kinase 1 gamma 1 (CSNK1G1) in Module 4 (Figure 3C). Among them, LATS1 was previously reported to be essential for male fertility.^[^ ^28^ ^]^ Deletion of PRKCD, a sperm acrosomal protein, disrupted fertilization and caused male subfertility.^[^ ^29^ ^]^ PRKG1, another acrosomal protein, is dispensable for male fertility.^[^ ^30^ ^]^ The functions of CSNK1G1 and TTBK2 are still elusive in spermatogenesis and male fertility.

### 2.4 CSNK1G1 Localized at Post Cap Phase Acrosome and is Important for Acrosome Biogenesis

**Figure 4:**
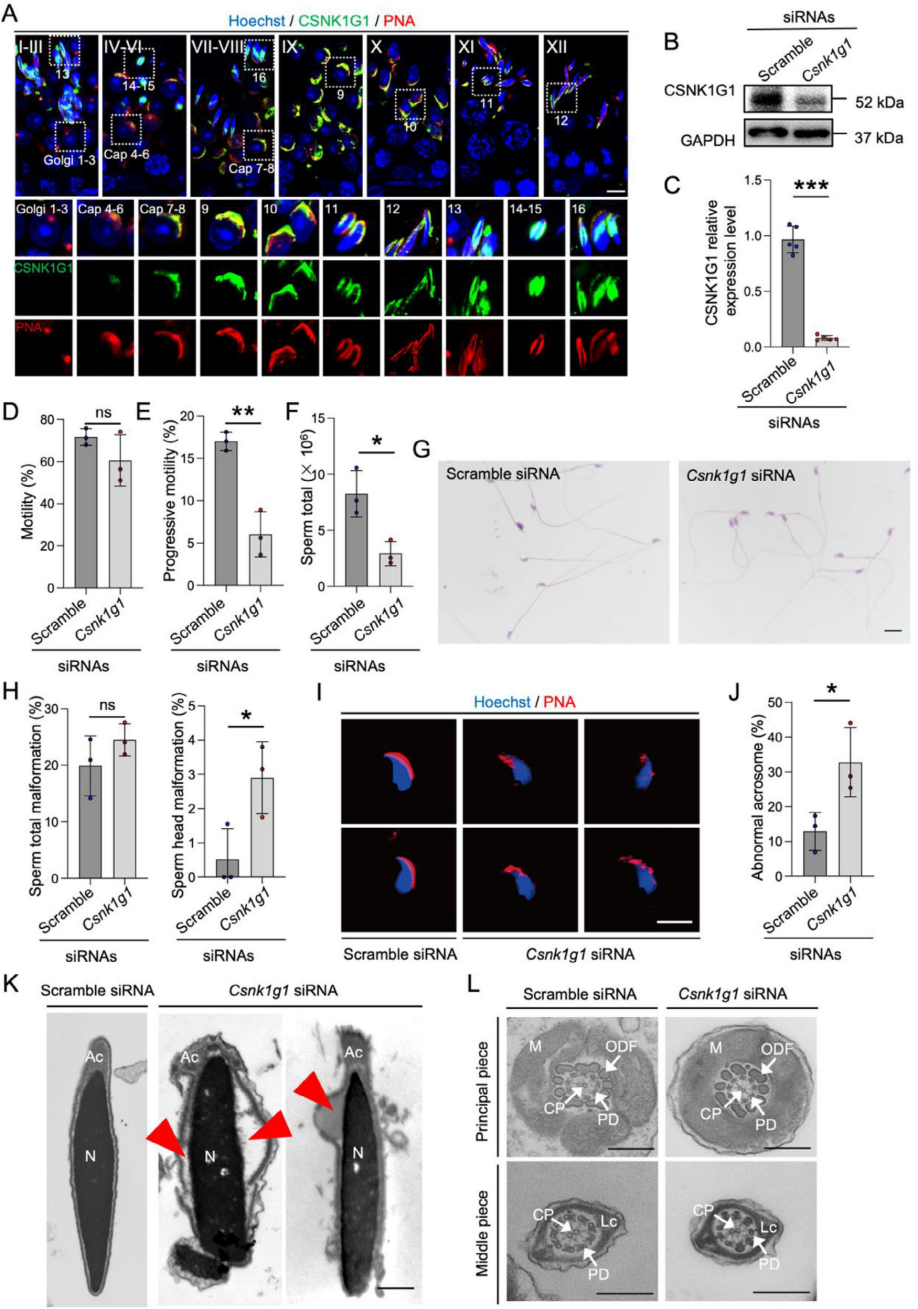
In Vivo Csnk1g1 knockdown disrupts acrosome development in spermatids A). Immunofluorescence of CSNK1G1 (green) in 12 stages (I to XII) of spermatogenesis and 16 steps (1‐16) of spermiogenesis in wildtype testis co‐stained with Hoechst (blue) and PNA (red). (Scale bar = 5 µm). B,C). Western blot (B) and statistical analysis (C) of efficiencies of Csnk1g1 siRNA in testis 72 h after injection, GAPDH was used as the loading control (n = 5, two‐tailed Student's t 25 This article is protected by copyright. All rights reserved. test, data are presented as mean ± SD, p < 0.0001). The relative abundance of IFT88 (C) was normalized to the intensity of GAPDH. D‐F. CASA of sperm parameters from cauda epididymides of four weeks after siRNA injection (n = 3, two‐tailed Student's t‐test), data are presented as mean ± SD, p = 0.2041 for D), p = 0.0028 for E), p = 0.0167 for (F). G,H). Representative images of Hematoxylin‐eosin (H&E) stained morphology (G) and statistics of total and head malformation rates (H, n = 3, two‐tailed Student's t‐test) of epididymal sperm under a light microscope from the scramble siRNA and Csnk1g1 siRNA groups. (Scale bars = 10 µm), statistics data are presented as mean ± SD, p = 0.2532 (left), p = 0.0401 (right). I,J). PNA‐staining(red) (I) and statistics of abnormal rates (J, n = 3, two‐tailed Student's *t*‐test, data are presented as mean ± SD, p = 0.0224) of sperm acrosomes in Csnk1g siRNA and scramble siRNA groups. (Scale bar = 10 µm.) K,L). Typical transmission electron microscopic images of sperm heads (K) and tails (L) from the scramble siRNA and Csnk1g1 siRNA groups. N, nucleus; Ac, acrosome; M, mitochondria; LC, longitudinal column; CP, central pair; PD, peripheral doublet; ODF, outer dense fiber. Red arrowhead, abnormal vacuoles in the acrosome. (Scale bar = 500 nm) ns, not significant, ^*^, p < 0.05; ^**^, p < 0.01; ^***^, p < 0.001.

CSNK1G1 is predominantly expressed in spermatids. To study the subcellular localization of CSNK1G1 in spermatids, we performed immunolocalization analysis, and found that CSNK1G1 was concentrated in acrosome starting from step 7–8 and throughout the rest of spermiogenesis, as indicated by co‐localization with PNA, an acrosome dye (**Figure** **4**A). The strong signal of CSNK1G1 after step 7–8 is consistent with our omics data showing the highest expression and kinase activity of CSNK1G1 in step 13–14 (module 4, Figure 3), indicating a possible important function in acrosome formation and function after cap phase.

To investigate the function of CSNK1G1, we knocked down *Csnk1g1* in vivo by microinjecting siRNA into rete testis. Western blot analysis showed CSNK1G1 protein level reduced by more than 50% compared to the scramble siRNA control after 48 h post‐injection (Figure 4B,C). Three weeks after siRNA injection, sperm derived from the caudal epididymis were collected for computer‐assisted sperm assessment (CASA), morphological, and ultrastructural analysis. The results showed no significant difference in overall sperm motility (Figure 4D), while progressive motility (Figure 4E) and sperm count were significantly decreased (Figure 4F). And H&E staining also showed no apparent abnormality in *Csnk1g1* siRNA group (Figure 4G,H).

PNA staining revealed a significantly increased proportion of irregularly shaped acrosomes in *Csnk1g* siRNA sperm, which were hardly observed in the control group (Figure 4I,J). To analyze the ultrastructural changes, we performed transmission electron microscopy (TEM) analysis. The results showed that in the control group, sperm had intact and normal acrosome covering condensed and elongated nuclei (Figure 4K), while the *Csnk1g*1 siRNA‐treated sperm exhibited abnormal vacuoles in the acrosome and discrete acrosome (Figure 4K). The middle or principal pieces of sperm flagella were also detected by TEM. Compared with the control group, there was no significant difference in the axonemal and peripheral structures of *Csnk1g*1 siRNA sperm (Figure 4L). Collectively, our results indicated CSNK1G1 is important for acrosome biogenesis.

### 2.5 *Ttbk2* Knockout in Germ Cells Causes Abnormal Flagella Biogenesis, Head Shaping, and Male Sterility

**Figure 5:**
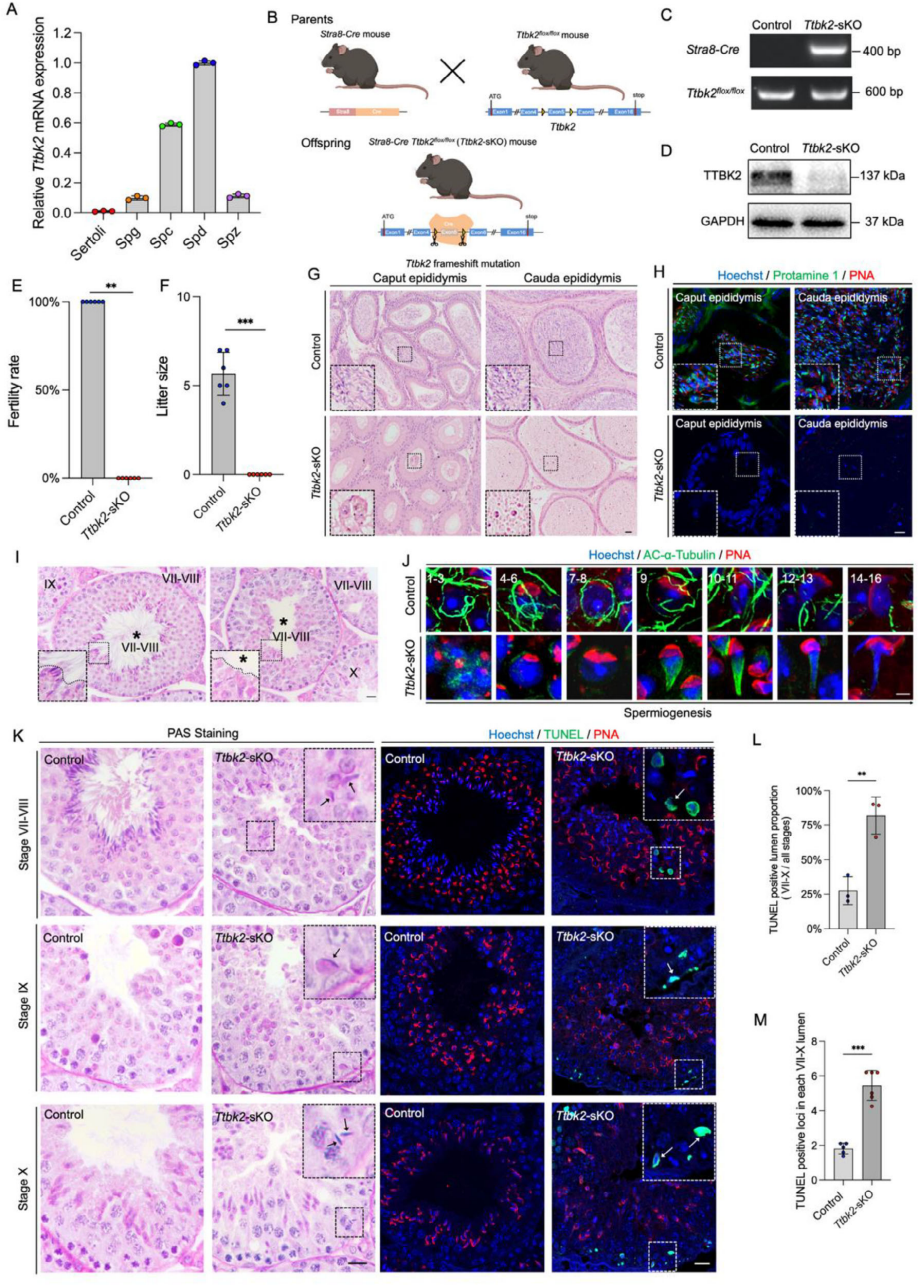
Germ cell‐specific Ttbk2 knockout causes abnormal flagella biogenesis, head shaping, and male sterility. A). Expression levels of Ttbk2 mRNA in five testicular cell types based on Soumillon et al.’s RNA‐seq. Ser, sertoli; Spg, spermatogonia; Spc, spermatocyte; Spd, spermatid; Spz, spermatozoa. B). Schematic diagram of germ cell‐specific deletion of exons 5 of Ttbk2 by Stra8 Cre‐mediated recombination. The Schematic diagram was created with BioRender.com, incorporating elements adapted from the template “rfdvhwn”, BioRender. Tianyu, Z. (2025) https://BioRender.com/rfdvhwn. C). Genotyping PCRs using Ttbk2flox/flox and Stra8‐Cre primers. D). 27 This article is protected by copyright. All rights reserved. Author Manuscript Western blot of TTBK2 in 8‐week Ttbk2‐sKO and Control testes. GAPDH was detected as an internal control. E‐F. Fertility rates (%) (E, n =  6, two‐tailed Student's *t*‐test, data are presented as mean ± SD, p = 0.0079) and litter sizes (F, n =  6 for each sample, two‐tailed Student's *t*‐test, data are presented as mean ± SD, p < 0.0001) of plugged wild‐type females after mating with 8‐week old Ttbk2‐sKO and Control males. G). H&E staining of the caput and cauda epididymis in Ttbk2 sKO and Control mice. (Scale bar = 20 µm). H). The immunofluorescence of Protamine 1 (green) in the caput and cauda epididymis with nuclei stained by Hoechst (blue) and acrosome stained by PNA (red). (Scale b;r = 20 µm) I). PAS staining of seminiferous tubules at stage VII‐VIII from Ttbk2‐sKO and Control male mice. (Scale bar = 20 µm) J). The immunofluorescence of AC‐α‐Tubulin (green) in spermatids at different steps with Hoechst (blue) and PNA (red). (Scale bar = 5 µm) K). PAS and TUNEL (green) staining of seminiferous tubules with Hoechst (blue) and PNA (red). (Scale bar = 20 µm) L). The percentage of TUNEL positive VII‐X lumens in the testicular sections from the Ttbk2‐sKO and Control mice (n =  3, two‐tailed Student's *t*‐test, data are presented as mean ± SD, p = 0.0051). M. TUNEL positive loci detected in each VII‐X lumen (n =  6 for Ttbk2‐sKO, n =  5 for Control, two‐tailed Student's t‐test, data are presented as mean ± SD, p < 0.0001). ^**^, p<0.01; ^***^, p<0.001.

Previous studies have shown that TTBK2 is essential for viability.^[^ ^31^ ^]^ In the testis, *Ttbk2* mRNA is predominantly expressed in spermatids and not expressed in Sertoli cells according to previous RNA‐seq data (**Figure** **5**A).^[^ ^27^ ^]^ To study the function of TTBK2 in spermiogenesis, we generated germ cell‐specific knockout *Stra8*‐Cre *Ttbk2^flox/flox^* mice (hereafter referred to as *Ttbk2*‐sKO) by Cre‐mediated removal of the exon 5 using flanking *loxP* sites (Figure 5B). Knockout of *Ttbk2* was validated by genotyping (Figure 5C), and the absence of TTBK2 protein level was observed in *Ttbk2*‐sKO testis (Figure 5D).

To elucidate the role of *Ttbk2* in male fertility and spermiogenesis, we conducted a phenotypic analysis of adult *Ttbk2*‐sKO mice aged 3–4 months, comparing them to age‐matched *Ttbk2^flox/flox^* control mice. We performed mating tests first, although *Ttbk2*‐sKO mice mating plugs were observed, no litters were obtained when *Ttbk2*‐sKO male mice were mating with wildtype females (Figure 5E,F). The generation of sperm is the basis of male fertility. We then examined epididymis by H&E staining. The results showed that the caput and cauda epididymis of *Ttbk2*‐sKO male mice were both devoid of sperm (Figure 5G,H), neither cells with sperm morphology (Figure 5G) nor Protamine 1 positive signals (Figure 5H) were observed. Further periodic acid‐Schiff (PAS) staining of *Ttbk2*‐sKO testis showed elongated spermatids without apparent flagella in stage VII‐VIII seminiferous epithelium (Figure 5I). To better show the flagella formation, we performed immunofluorescence of acetyl‐α‐Tubulin (AC‐α‐Tubulin) in spermatid cells (Figure 5J) and mouse testicular sections (Figure 4C, Supporting Information). The results showed the flagella were absent in all developing steps of spermatids, indicating essential functions of TTBK2 in the generation of flagella (Figure 5J; Figure S4C, Supporting Information). Furthermore, retaining of step 16 elongated spermatids in stage IX and X (Figure 5K) was observed in *Ttbk2*‐sKO, indicating delayed release at stage VIII. Further TUNEL assay showed TUNEL positive signal in hook head‐shaped spermatids, indicating apoptosis of the retained spermatids (Figure 5K–M).

**Figure 6:**
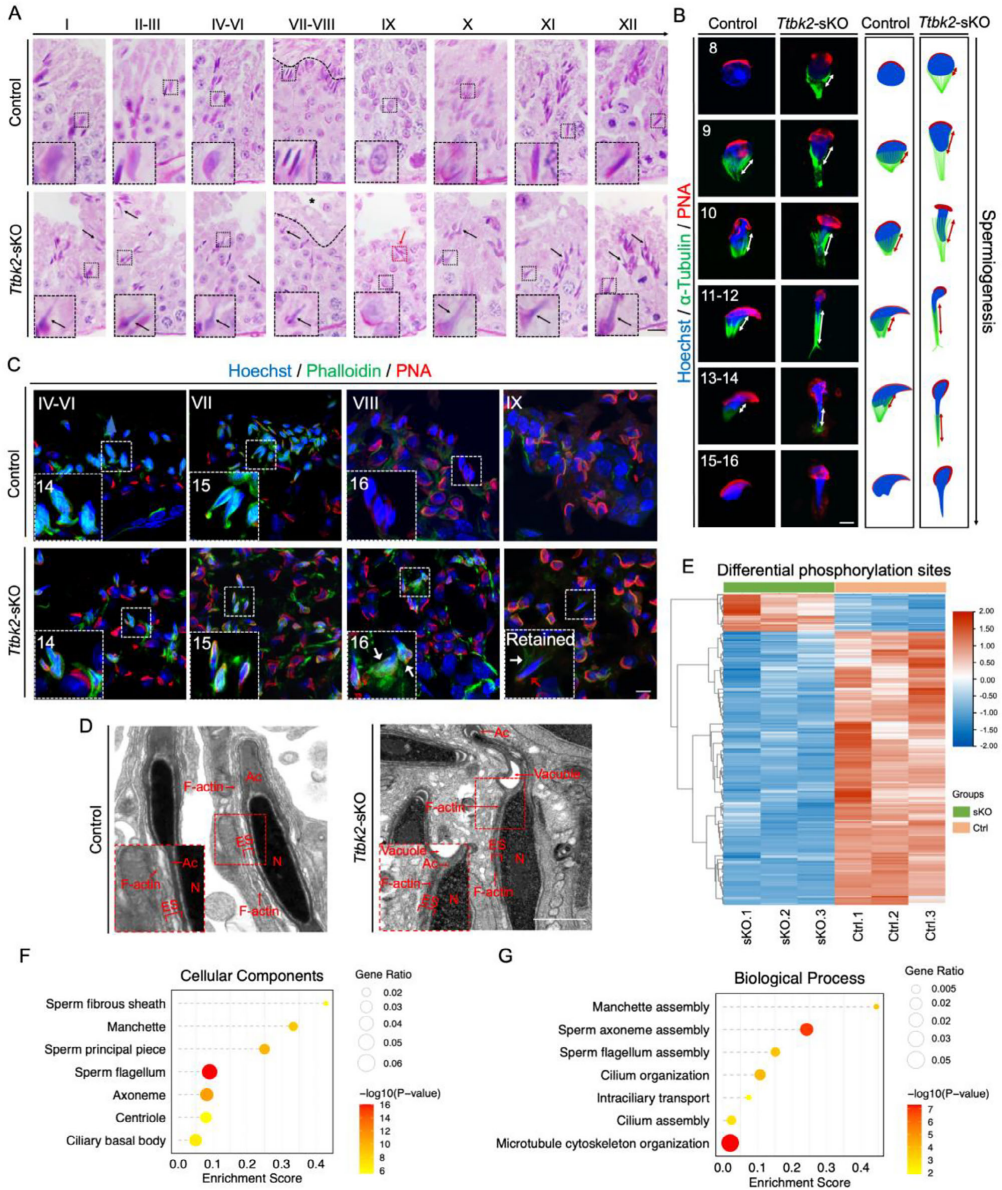
The Ttbk2‐sKO spermatids exhibit abnormal morphologies and protein levels. A). PAS staining of seminiferous tubules at different stages in Ttbk2‐sKO and Control mice. (Scale bar = 20 µm). B). Spermatids were stained with Hoechst (blue), PNA (red), and α‐tubulin (green, marker of manchette) from steps 8 to 16 in Ttbk2‐sKO and Control mice. (Scale bar = 5 µm). C). Elongated spermatids from stages IV to IX from Ttbk2‐sKO and Control seminiferous tubules stained with Hoechst (blue), PNA (red), and phalloidin (green). (Scale bar = 5 µm). D). The TEM ultra‐structures of Ttbk2‐sKO and Control spermatids. (Scale bar = 1 µm). N, nucleus; Ac, acrosome; ES, ectoplasmic specialization. E. Heatmap of differential phosphorylation sites between Ttbk2‐sKO and Control male mice. GO terms of CCs (F) and BPs (G) enriched in proteins with downregulated phosphorylation sites in Ttbk2‐sKO testis.

In addition to sperm flagella defects, PAS staining of *Ttbk2*‐sKO testis also showed abnormal club‐shaped nuclear morphology of spermatids since step 10, contrary to the hook‐shaped nuclei in the control elongated spermatids (**Figure** **6**A). Manchette, a microtubular based structure parallel to the spermatid nucleus, facilitate nuclear elongation. To evaluate the structure of manchette, we performed immunofluorescence of α‐Tubulin, and found abnormal elongation of α‐Tubulin signals in *Ttbk2*‐sKO spermatids (Figure 6B). The club‐shaped nuclei shape is caused by abnormal manchette structure during spermiogenesis.

No mature spermatozoa were found in the *Ttbk2*‐sKO epididymis (Figure 5F), however, spermiogenesis of *Ttbk2*‐sKO mice was not arrested, with spermatids reaching step 16. Spermatid release requires degradation of the apical ectoplasmic specialization (ES) junction, an F‐actin‐based structure anchoring elongating spermatids to Sertoli cells.^[^ ^32^ ^]^ In stage VIII control seminiferous tubules, F‐actin signals weakened, indicating disassembly of ES junction. However, F‐actin was retained at the apical region of spermatids till stage IX in *Ttbk2*‐sKO seminiferous tubules (Figure 6C). TEM analysis of *Ttbk2*‐sKO testis confirmed that the apical ES remained intact, extending along with the acrosome in the elongated spermatids, while F‐actin bundles were fully removed in the control elongated spermatids (Figure 6D). These findings suggested that the absence of sperm in *Ttbk2*‐sKO epididymis is due to spermiation deficiency.

### 2.6 TTBK2 Regulates IFT88 Phosphorylation and Stability During Sperm Flagella Formation

To investigate the downstream phosphorylation proteins regulated by TTBK2 kinase in spermiogenesis, we performed quantitative phosphoproteomic profiling of *Ttbk2*‐sKO testicular proteins using TMT labeling with Ti‐IMAC enrichment and LC‐MS/MS analysis. We quantified 17,716 phosphorylation sites corresponding to 4,641 phosphorylated proteins (Table S6a, Supporting Information), with 629 differentially type I phosphorylated sites (localization probability > 0.75, Intensity_1 > 0, fold change > 1.5, p < 0.05) corresponding to 415 proteins between *Ttbk2*‐sKO and control groups after normalization to protein abundance levels. Among these, 558 phosphorylation sites (88.7%, 558/629) corresponding to 369 phosphoproteins were downregulated, whereas only 71 sites among 52 phosphoproteins were upregulated (Figure 6E; Table S6b, Supporting Information). The majority of the differentially regulated phosphorylation sites were downregulated, indicating the important effects of kinase activity loss after *Ttbk2* knockout. We performed gene ontology (GO) enrichment analysis of these phosphoproteins downregulated in *Ttbk2*‐sKO testis at phosphorylation level. The cellular component (CC) analysis showed significant enrichment in sperm flagellum, sperm FS, and ciliary basal body, etc. (Figure 6F; Table S7a, Supporting Information). The biological process (BP) analysis highlighted enrichment in Manchette assembly, sperm flagella assembly, sperm axoneme assembly, cilium organization, and intraciliary transport, etc. (Figure 6G; Table S7b, Supporting Information), underscoring the important roles of TTBK2 kinase‐mediated phosphorylation in sperm flagella formation.

**Figure 7:**
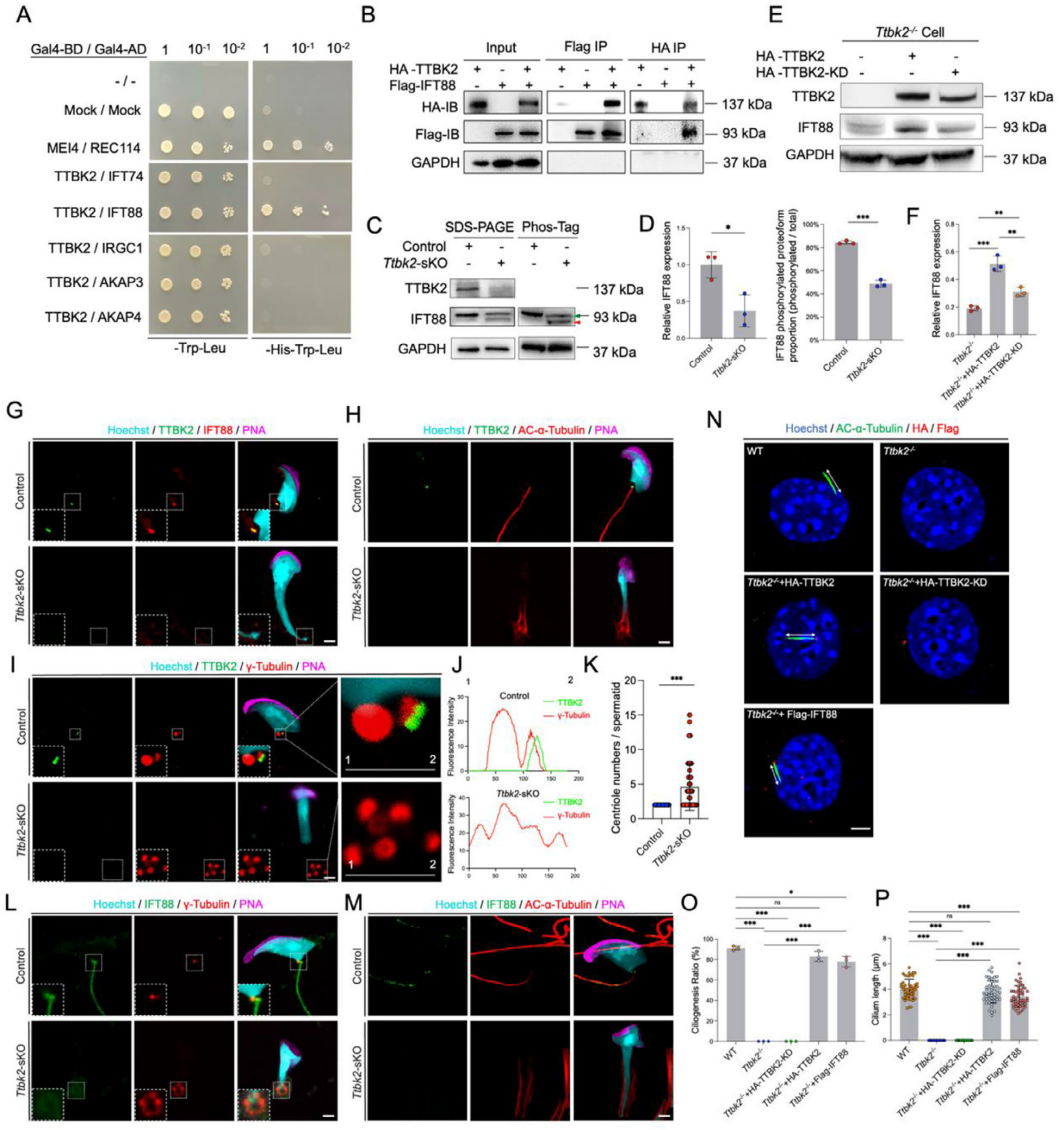
TTBK2 regulates phosphorylation and stability of IFT88 to facilitate sperm flagella formation. A). Y2H assay of interactions between TTBK2 and candidate interacting proteins. MEI4 and REC114 were used as positive controls, and empty vectors of pGBKT7 and pBAKT7 were used as negative controls. B). Reciprocal co‐IP assay of interaction between HA TTBK2 and Flag‐IFT88 in HEK293T cells. C). TTBK2 and IFT88 immunoblotting of Ttbk2 sKO and Control testicular lysate samples in SDS‐PAGE (Left) and PhosTM‐Tag (Right) immunoblot. Green arrowhead, band of phosphorylated IFT88 proteoform; Red arrowhead, band of non‐phosphorylated IFT88 proteoform. D. Quatifications of (C) (n = 3, two‐tailed Student's t‐test), data are presented as mean ± SD, p = 0.0181 (left), p < 0.0001 (right), IFT88 intensity (left), and IFT88 phosphorylated proteoform proportion (right) in Ttbk2‐sKO and Control testicular lysates were quantified. The relative abundance of IFT88 (left) was normalized to the intensity of GAPDH. And the abundance of the phosphorylated proteoform (right) was normalized to that of the total proteoform. E,F). Expression levels (E) and quantification (F, n = 3, two‐tailed Student's *t*‐test, data 30 This article is protected by copyright. All rights reserved. Author Manuscript are presented as mean ± SD, p = 0.0007 for Ttbk2‐/‐ vs. Ttbk2‐/‐ + HA‐TTBK2, p = 0.0058 for Ttbk2 /‐ vs. Ttbk2‐/‐ + HA‐TTBK2‐KD, p = 0.0056 for Ttbk2‐/‐ + HA‐TTBK2 vs. Ttbk2‐/‐ + HA‐TTBK2 KD) of TTBK2, IFT88 proteins in Ttbk2‐/‐ NIH3T3 cells with overexpression of HA‐TTBK2 or HA TTBK2‐KD (D163A) with GAPDH as a loading control. The relative abundance of IFT88 (F) was normalized to the intensity of GAPDH. G‐J. Immunofluorescence analysis of TTBK2 (green) and IFT88 (red) (G), AC‐α‐Tubulin (red) (H), and γ‐Tubulin (red) (I) in Ttbk2‐sKO and Control testis with Hoechst (cyan) and PNA (magenta). Overlapping fluorescence intensities between TTBK2 and γ‐Tubulin were shown (J). (Scale bar = 3 µm). K. The number of γ‐Tubulin‐positive centrioles compared between the Ttbk2‐sKO and Control (n = 51 for three Control samples, n = 89 for three Ttbk2‐sKO samples; two‐tailed Student's t‐test, data are presented as mean ± SD, p < 0.0001). L,M). Immunofluorescence of IFT88 (green) and γ‐Tubulin (red) (L), AC‐α‐Tubulin (red) (M) in Ttbk2‐sKO and Control testis with Hoechst (cyan) and PNA (magenta). (Scale bar = 3 µm). N–P). Ciliogenesis ratio and cilia length in Ttbk2‐/‐ cells with or without HA‐TTBK2, HA‐TTBK2‐KD, and Flag‐IFT88 overexpression based with anti‐Ac‐α‐tubulin (green), anti‐HA (red), anti‐Flag (red) antibodies, and Hoechst (blue). Cilia were indicated by double‐head arrows. (Scale bar = 3 µm) (N). The quantification results of cilliogenesis ratio (O, 3 biological replicates in each group, two‐tailed Student's t‐test, data are presented as mean ± SD, p < 0.0001 for WT vs. Ttbk2‐/‐, p < 0.0001 for WT vs. Ttbk2‐/‐ + HA‐TTBK2‐KD, p = 0.06 for WT vs. Ttbk2‐/‐ + HA‐TTBK2, p = 0.0149 for WT vs. Ttbk2‐/‐ + Flag‐IFT88, p < 0.0001 for Ttbk2‐/‐ vs. Ttbk2‐/‐ + HA‐TTBK2, p < 0.0001 for Ttbk2‐/‐ vs. Ttbk2‐/‐ + Flag‐IFT88) and cilia length (P, 3 biological replicates in each group, n = 50 for each group, two‐tailed Student's *t*‐test, data are presented as mean ± SD, p < 0.0001 for WT vs. Ttbk2‐/‐, p < 0.0001 for WT vs. Ttbk2‐/‐ + HA‐TTBK2‐KD, p = 0.1485 for WT vs. Ttbk2‐/‐ + HA‐TTBK2, p = 0.0003 for WT vs. Ttbk2‐/‐ + Flag‐IFT88, p < 0.0001 for Ttbk2‐/‐ vs. Ttbk2‐/‐ + HA‐TTBK2, p < 0.0001 for Ttbk2‐/‐ vs. Ttbk2‐/‐ + Flag‐IFT88). ns, not significant; ^*^, p < 0.05; **, p < 0.01; ***, p<0.001.

The downregulated phosphorylation sites after TTBK2 deletion may be direct or indirect downstream phosphoproteins. To identify potential direct substrates of TTBK2, we first performed differential phosphoproteomic analysis comparing control and *Ttbk2*‐sKO testes. Then we prioritized downregulated phosphorylation sites based on functional relevance to the flagellar defects observed in *Ttbk2*‐deficient sperm. Several downregulated phospho‐proteins with sperm flagella phenotype or localization were identified, including IFT74,^[^ ^33^ ^]^ IFT88,^[^ ^34^ ^]^ IRGC1,^[^ ^35^ ^]^ AKAP3^[^ ^36^ ^]^ and AKAP4,^[^ ^37^ ^]^ etc. Yeast two‐hybrid (Y2H) analysis of the interaction of TTBK2 with five proteins showed that TTBK2 interacted with IFT88 (**Figure** **7**A). We further performed reciprocal co‐IP analysis (Figure 7B) and confirmed the interaction between TTBK2 and IFT88. To validate phosphorylation of IFT88 by TTBK2, we performed phosphorylation‐induced gel mobility shift assay with CCDC92, a previously reported substrate,^[^ ^38^ ^]^ as a positive control (Figure S5A, Supporting Information). The results showed that in control testicular proteins, but not in *Ttbk2*‐sKO, IFT88 exhibited a shift in the Phos‐tag gel, indicating that IFT88 phosphorylation is TTBK2‐dependent (Figure 7C).

IFT88 is a ciliary transport protein responsible for the forward transport of proteins in cilia. TTBK2 might regulate the function of IFT88 to regulate flagella formation. We performed Western blot analysis of the expression of IFT88, and found significantly reduced expression of IFT88 in *Ttbk2*‐sKO testes compared with controls (Figure 7C,D). To confirm the regulation of IFT88 by TTBK2, we generated a *Ttbk2* knockout cell line (*Ttbk2* ^−/−^) using CRISPR‐Cas9 technology, and overexpressed HA‐TTBK2 or kinase‐dead HA‐TTBK2 (D163A). Western blot analysis revealed that *Ttbk2* knockout reduced IFT88 protein level in *Ttbk2* ^−/−^ cells (Figure S5B,C, Supporting Information). The IFT88 expression could be rescued by overexpressing HA‐TTBK2 but not kinase‐dead HA‐TTBK2 in *Ttbk2* ^−/−^ cells (Figure 7E,F). The above results indicate that TTBK2 kinase‐mediated IFT88 phosphorylation is important for protein stability of IFT88.

### 2.7 IFT88 Overexpression Can Partially Rescue the Ciliogenesis Defects Caused by TTBK2 Deletion

To study the regulation of IFT88 by TTBK2 at the subcellular localization level during flagellar formation, we performed co‐immunofluorescent analyses. The co‐localization analysis of IFT88 and TTBK2 revealed that IFT88 is colocalized with TTBK2 in spermatids, and the fluorescent signal of IFT88 decreased after *Ttbk2* deletion (Figure 7G). The elongation of flagella axoneme starts at the centriole.^[^ ^24^ ^]^ The above results showed that flagella were absent in *Ttbk2*‐sKO spermatids. To investigate whether *Ttbk2* regulates the centrioles and formation of flagella during spermiogenesis, we stained the flagella and centrioles with anti‐AC‐α‐Tubulin and anti‐γ‐tubulin, and performed co‐stained TTBK2 and IFT88 in *Ttbk2‐*sKO spermatids (Figure 7H,I,L,M). The results suggested that TTBK2 is primarily located at the base part of AC‐α‐Tubulin positive flagella, and colocalize with γ‐tubulin at the centriole (Figure 7H–J), while IFT88 is not only localized at centriole, but also distributed along the entire flagella. Moreover, we found that all the spermatids had two centrioles in the control group, while the number of centrioles significantly increased in *Ttbk2*‐sKO spermatids (Figure 7I,K), indicating the centriole abnormalities after *Ttbk2* deletion.

TTBK2 deletion caused IFT88 decrease and defects of flagellar formation. To evaluate the ability of IFT88 to functionally rescue flagellar formation after TTBK2 deletion, we first generated *Ttbk2* ^−/‐^ cells, which underwent serum starvation for two days to induce ciliary development. The results showed that the *Ttbk2* ^−/‐^ cells displayed impaired ciliary induction, in contrary to the normal cilia development in wild‐type control cells. Subsequently, we overexpressed HA‐TTBK2, kinase‐dead HA‐TTBK2 or Flag‐IFT88 to evaluate the effects to rescue ciliary development in *Ttbk2* ^−/‐^ cells. The results showed that TTBK2 kinase dead cannot rescue the ciliogenesis deficiency, which indicated that the kinase activity of TTBK2 is essential for ciliogenesis (Figure 7N). Ciliogenesis statistics showed that cilia were absent in *Ttbk2* ^−/‐^ cell line, it could be rescued by HA‐TTBK2 overexpression and partially rescued by Flag‐IFT88 overexpression (Figure 7O). Further length statistics analysis showed that the cilia length could also be rescued by HA‐TTBK2 overexpression, and partially rescued by Flag‐IFT88 overexpression (Figure 7P). Altogether, the TTBK2 kinase‐dependent IFT88 stability is important for ciliogenesis.

## 3 Discussion

Phosphorylation is known to be important for spermiogenesis. Li et al. performed phosphoproteomic analysis of mixed spermatids.^[^ ^9^ ^]^ However, the temporal dynamics of phosphoproteome during spermiogenesis is still not known. Here, we isolated step‐specific spermatids from spermatogenesis‐synchronized testis and characterized a high‐resolution phosphoproteomic atlas. After multi‐layered screening pipeline to prioritize functionally relevant kinases, TTBK2 and CSNK1G1 were found to be important for spermiogenesis. These findings not only provide mechanistic insights into phosphorylation‐mediated regulation during sperm morphogenesis but also establish a framework for functional exploration of stage‐specific kinase signaling in male germ cell differentiation.

Our study focused on four developmental windows of spermiogenesis with morphogenetic changes. Step 1–2 represent the onset of post‐meiotic development and initial flagellar formation; step 3–4 mark early acrosome biogenesis; step 5–6 coincide with microtubule cytoskeletal rearrangement for manchette assembly; and step 13–14 encompass cytoplasmic reduction and histone‐to‐protamine replacement. Steps 1–2 and 3–4 showed only modest differences; more pronounced divergence emerged between steps 3–4 and 5–6, coinciding with cytoskeletal remodeling and manchette formation. Interestingly, both proteomic and phosphoproteomic analyses revealed only modest differences between step 1–2 and 3–4 spermatids, particularly within Module 1. The spermatids were isolated from RA‐synchronized testes, with only a narrow range of developmental stages in each time point. Morphological criteria and PNA staining patterns clearly distinguish step 3–4 spermatids from earlier stages, as shown in Figure S1 (Supporting Information). These combined lines of evidence support the biological authenticity of the observed similarities, rather than technical artifacts, and highlight a continuous yet temporally regulated phospho‐signaling landscape during early spermatid development.

Leveraging synchronized spermatogenesis and sensitive quantitative phosphoproteomics, we quantified 4,466 phosphoproteins and 13,054 phosphosites across the four steps, including 2,059 newly identified sites. These dynamic phosphoproteins are enriched in pathways associated with acrosome, flagellum, and head development. Kinase‐substrate enrichment analysis identified 27 kinases with stage‐regulated substrate phosphorylation, and functional analysis uncovered important roles of CSNK1G1 in acrosome biogenesis and TTBK2 in stabilizing IFT88 to support flagellar assembly.

We found that the proteins differentially regulated at the phosphorylation level are enriched in chromatin, acrosome, flagella, and condensed nuclei. Dynamic phosphorylation of chromatin‐associated proteins likely plays regulatory role in facilitating the dramatic reorganization of chromatin structure transition. The manchette is typically observed from step 7–8 onward. But cytoskeletal remodeling and microtubule organization events that precede manchette formation occur as early as step 5–6.^[^ ^39^ ^]^ Step 7–12 elongating spermatids co‐exist with haploid round spermatids or secondary spermatocytes, which have similar buoyant densities, and cannot be purified using STA‐PUT separation. We observed peak phosphorylation of the manchette‐associated proteins, including RIMBP3, during step 5–6 (Figure 1J). IFT components are recruited and assembled at the basal body primarily at the initiation stage of ciliogenesis, ensuring efficient transport and ciliary formation,^[^ ^40^ ^]^ the higher phosphorylation levels of IFT‐B complex including IFT57‐pT293, IFT‐pS14, IFT74‐pS300, IFT81‐pS89, and IFT22‐pS107 in step 1–2 indicate important phosphorylation regulation of IFT‐B in the assembly of IFT complex at the onset of flagella biogenesis. Meanwhile, phosphorylation of IFT‐A, IFT‐B, dynein, and myosin proteins of IFT machineries, IFT81‐pS89, IFT122‐pS820, IFT122‐pS225, DNAH6‐pS3210, and MYO9B‐pS1935, all showed relatively high phosphorylation levels in step 5–6. Their active phosphorylation might also be involved in the transportation of microtubule‐based manchette.^[^ ^41^ ^]^ The microtubule‐based intra‐manchette transportation (IMT) relies on IFTs, kinesins, and dynein.^[^ ^41^ ^]^ Although dynamically regulated at the phosphorylation levels, their protein levels remained unchanged during spermiogenesis. To further elucidate the regulation of flagellar formation and manchette regulation, it is important to characterize the functions of their phosphorylation. Phosphorylation is an important layer of regulation during spermiogenesis, different from the expression level.

By integrating phosphorylation module dynamics, protein abundance trends, and spermatogenic cell‐type transcriptomes, we prioritized five kinases (PRKG1, LATS1, TTBK2, PRKCD, and CSNK1G1) with potential regulatory roles in spermiogenesis, including known fertility‐related kinases (LATS1, PRKCD), fertility‐dispensable kinase (PRKG1), and uncharacterized candidates (TTBK2, CSNK1G1). Notably, both TTBK2 and CSNK1G1 exhibited dynamic regulation of their substrates in spermatids and showed consistent protein and mRNA enrichment in post‐meiotic germ cells. We further found that CSNK1G1 kinase is an acrosomal protein. Although in vivo knockdown of *Csnk1g1* led to CSNK1G1 protein reduction only by ≈50% in spermatids, we observed severe acrosomal defects. This suggests a CSNK1G may function in a dosage‐dependent manner, where a partial reduction in CSNK1G1 crosses a functional boundary required for maintaining acrosomal assembly and integrity.

Among the significant kinases in kinase substrate phosphorylation network analysis, we found that TTBK2 was essential for male fertility. TTBK2 is a serine/threonine kinase with a highly conserved kinase domain at its N‐terminal region.^[^ ^42^ ^]^ Previous knockout studies have shown that TTBK2 is essential for cilliogenesis and Sonic hedgehog (Shh) signaling—a pathway crucial for embryogenesis, resulting in holoprosencephaly, abnormal neural tube and limb development, and embryonic lethality after *Ttbk2* knockout. Although sperm flagella and cilia are believed to have a similar “9+2” arrangement of microtubule structures and flagellar transport and assembly mechanisms.^[^ ^43^ ^]^ Our study found that TTBK2 is not only essential for sperm flagellum, but also important for manchette structure. The manchette is composed of microtubule bundles parallel to the nuclear membrane and is a key structure for shaping the head of sperm cells.^[^ ^44^ ^]^ Germ cell‐specific deletion of *Ttbk2* led to abnormally extended manchette, resulting in abnormal head shaping. TTBK2 has germ cell‐specific functions in spermiogenesis.

During spermiogenesis, we found that TTBK2 phosphorylates IFT88 to regulate sperm flagellar formation. Notably, three adjacent phosphorylation sites on IFT88 (pS745, pS748, and pS750) were significantly downregulated in *Ttbk2*‐sKO testes. This clustering may suggest conformational or interaction‐related regulation, potentially contributing to the observed reduction in IFT88 protein level. While further studies are needed, these data raise the possibility that TTBK2‐mediated phosphorylation may stabilize IFT88 during spermatid development. Previous studies showed that TTBK2 can phosphorylate CEP83 to trigger the removal of CP110, and regulate the initiation of ciliation.^[^ ^45^, ^46^ ^]^ IFT88^[^ ^34^ ^]^ is localized at manchette and spermatid flagella and is essential for spermiogenesis and male fertility. Its loss causes sperm flagella and manchette defects.^[^ ^34^ ^]^ Kinases can phosphorylate of their substrates to regulate their function or stability.^[^ ^47^ ^]^ We found that TTBK2 can interact with and regulate phosphorylation and expression level of IFT88 in *Ttbk2*‐sKO mice testis in a kinase activity‐dependent manner. Thus, TTBK2 regulated manchette function and flagella formation via phosphorylation of IFT88. Also, reduced acetylated α‐tubulin signal in *Ttbk2*‐sKO spermatids was observed, which suggests impaired flagellar assembly. While no direct change in acetyltransferases aTAT1 phosphorylation was observed in the knockout mice, aTAT1 is phosphorylated during spermiogenesis and clusters in Module 3, aligning with flagellum development. This implies TTBK2 may regulate acetylation indirectly via pathways involving aTAT1.

Although TTBK2's canonical role is tightly associated with the assembly of cilia. However, our findings reveal an unexpected function of TTBK2 in regulating manchette formation during spermiogenesis—a structure unique to elongating spermatids, served as a transient microtubule‐based structure that facilitates nuclear shaping and serves as a scaffold for protein trafficking during sperm head morphogenesis and is distinct from classical cilia. That TTBK2 regulates this cytoskeletal structure suggests a broader role of TTBK2 beyond canonical ciliogenesis, possibly through modulation of microtubule dynamics or cargo transport mechanisms conserved across different cell types.

From a translational perspective, our identification of germ cell‐enriched kinases with key roles in sperm flagella and acrosome biogenesis raises the possibility that kinases such as CSNK1G1 may serve as candidate targets for non‐hormonal male contraception. However, TTBK2 is also essential for primary cilia function in neurons, particularly critical to the survival of cerebellar Purkinje cells,^[^ ^45^ ^]^ and thus may not be suitable for systemic inhibition. Future studies are warranted to evaluate the safety, specificity, and reversibility of targeting kinases in the context of male fertility regulation.

While our study uncovered stage‐specific phosphorylation programs and functionally validated key kinases such as TTBK2 and CSNK1G1 in sperm morphogenesis, certain limitations remain. Although we associated dynamic phosphorylation patterns with functional processes, direct causality remains to be tested by targeted phosphosite mutagenesis and rescue experiments. Additionally, siRNA knockdown strategies may carry off‐target risks. However, we employed a well‐established in vivo testicular delivery protocol,^[^ ^48^, ^49^ ^]^ and a published transcriptomic analysis^[^ ^27^ ^]^ indicates that *Csnk1g1* is predominantly expressed in spermatids, with minimal expression in Sertoli cells. This expression pattern reduces the likelihood of off‐target effects in Sertoli cells and supports that the observed phenotypes primarily originate from spermatid‐target gene knockdown.

## 4 Conclusion

In brief, we performed systemic quantitative phosphoproteomics profiling of mouse spermatids undergoing 4 different developing steps, and found enrichment of differentially regulated phosphoproteins in key processes involved in spermiogenesis. Kinase substrate phosphorylation network analysis showed significant kinases, including CSNK1G1, whose knockdown caused defects in acrosome biogenesis. Another significant kinase, TTBK2, was found to be essential for the phosphorylation and stability of IFT88, and its germ cell‐specific deletion led to sperm flagella development and sperm head shaping defects. Our research demonstrated the essential roles of dynamic kinase activities during spermiogenesis and provided a rich resource for further investigations of the mechanisms of spermiogenesis.

## 5 Experimental Section

### Spermatogenesis Synchronization

Spermatogenesis synchronization was performed according to previously published protocols.^[^ ^16^, ^17^ ^]^ Briefly, male C57BL/6J mice from P2 to P8 were pipette fed with WIN 18,446 (Abmole, Beijing, China), in a ratio of 100 µg WIN 18,446/1 g body weight per day. Differentiation of spermatogonia was reinitiated on day 8 after WIN 18,446 treatment, by intraperitoneal injection of retinoic acid (RA) (Sigma, St. Louis, MO), with the dosage of 35 µg RA/1 g body weight. According to the previously reported timeline of spermatid development in synchronized testis,^[^ ^16^ ^]^ step 1–2 spermatids on P27 testes, step 3–4 on P28/34, step 5–6 on P30.5, and step 13–14 on P35.5 were purified by STA‐PUT separation. At P34, step 11–12 elongating spermatids begin to appear together with step 3–4 spermatids, but step 3–4 spermatids can still be isolated to achieve over 90% purity by STA‐PUT separation. Therefore, step 3–4 spermatids from P28 and P34 were pooled for subsequent analyses.  The synchronization efficiency was determined by PAS staining and PNA/AC‐α‐Tubulin immunoflourescence analyses (Figure S1, Supporting Information). All animal procedures were approved by the Institutional Animal Care and Use Committees of Nanjing Medical University (IACUC‐2105055‐2).

### STA‐PUT Isolation of Spermatids

Spermatid at steps 1–2, 3–4, 5–6, and 13–14 were isolated by the STA‐PUT method described as previously reported.^[^ ^18^, ^50^ ^]^ Synchronized testicular tissues were decapsulated, digested with 1 mg/ml collagenase IV (Invitrogen, Waltham, MA) at 37 °C for 10 min, and centrifuged at 600 g for 10 min to collect seminiferous tubules. Seminiferous tubules were digested with 1 mg/ml DNase I (Sangon‐Biotech, Shanghai, China) dissolved in 0.25% trypsin (Gibco, Grand Island, NY) in 37 °C for 8 min, and terminated with an equal volume of Dulbecco's Modified Eagle Medium (DMEM) containing 10% Fetal Bovine Serum (Gibco, Grand Island, NY), the cells was filtered with 100 µm nylon strainer (Corning, Corning, NY), and put into the cell loading chamber; a 2% and 4% bovine serum albumin (BSA) (Sigma, St. Louis, MO) prepared in DMEM were used to generate a 2–4% density gradient of BSA solution in the sedimentation chamber. After 2.25 h of precipitation, 120 consecutive fractions were collected and analyzed, with step 1–2 spermatids from fractions 36–46, step 3–4 from fractions 53–63, step 5–6 from fractions 49–59, and step 13–14 from fractions 93–109. The spermatids were stained with Hoechst (Molecular Probes, Eugene, OR) and distinguished under an inverted light microscope (Nikon ECLIPSE Ti2, Tokyo, Japan), by nuclei morphology and cell diameters. The collected spermatids were stained with PNA (Vector Laboratories, Newark, CA) and Hoechst, and their purity and developing stages were evaluated by the morphologies of nuclei and acrosomes.

### PAS Staining

Mouse testicular tissues were fixed with modified Davidson's fixation fluid (mDF). After 48 h fixation at room temperature, the tissues were embedded in paraffin and sectioned at 5 µm thickness. The sections were dewaxed with xylene at 37 °C for 30 min, rehydrated by 100%‐70% gradient descending concentrations of ethanol, stained with PAS (Solarbio, Beijing, China), and counterstained with hematoxylin to label the nuclei, dehydrated with 100%–70% gradient ascending concentrations of ethanol, and sealed with neutral balsam. Images were taken with ECLIPSE Ni‐E (Nikon, Tokyo, Japan).

### Immunofluorescence Staining and Image Acquisition

For immunofluorescence, the sections were subjected to heat‐induced antigen retrieval with citrate buffer (1.8 mm citric acid, 8.2 mm sodium citrate, pH 6.0), washed with PBS, and fixed in 4% Paraformaldehyde (PFA). The samples were blocked with 5% BSA at room temperature for 2 h, and then overnight incubated with primary antibodies at 4 °C. After washing with PBS, samples were incubated at room temperature for 2 h with the secondary antibody (Abcam, Cambridge, UK), PNA (Vector Laboratories, Newark, CA), and Hoechst (Thermo Fisher, Waltham, MA), then mounted under a coverslip using antifade mounting reagent (Invitrogen, Waltham, MA).

For triple staining of seminiferous tubule squashes, samples were fixed with 4% PFA for 30 min and blocked with 5% BSA for 2 h. For co‐staining with TTBK2, IFT88, and mouse (acetylated tubulin, γ‐tubulin) primary antibodies, all antibodies were co‐incubated overnight at 4 °C, followed by species‐specific secondary antibodies and PNA for 2 h at room temperature. Samples were mounted in an antifade medium for imaging. Sequential staining was used to achieve co‐staining of TTBK2 and IFT88. TTBK2 was applied first, followed by anti‐rabbit secondary and re‐blocking. IFT88 was then incubated overnight, and the second secondary antibody was added with PNA before mounting and imaging.

All images were captured on a Leica TCS SP8 confocal microscope using either a 40× oil‐immersion objective or a 63× oil‐immersion objective (Leica, Wetzlar, Germany). For all channels, laser power was set between 1–3.5% of maximum; detector gain was 500–800 HV, the pinhole was set to 1 AU, acquisition was 1,024×1,024 pixels or 2,048×2,048 pixels, scan speed 400 Hz, line averaging of 4, and digital zoom of 1.0 × ‐6.0×.

### *Ttbk2* Conditional Knockout (cKO) Mouse Generation

*Ttbk2^flox/flox^* mice were obtained from the Center for Animal Model, Soochow University (CAM‐SU GRC), originally generated by the EUCOMM project. *Stra8*‐Cre mice was gifted from Minghan Tong Lab.^[^ ^51^ ^]^ *Ttbk2^flox/flox^* were crossed with *Stra8*‐Cre mice to generate male germ cell–specific knockout mice. All mice used in this study were kept in C57BL/6J genetic background and housed under specific pathogen‐free (SPF) conditions with standard food and hypochlorous weak‐acid water. All animal procedures were approved by the Institutional Animal Care and Use Committees of Nanjing Medical University (IACUC‐1810007).

### Y2H Assay

Y2H assay was performed as previously described.^[^ ^52^ ^]^ We cloned mouse *Ttbk2* into pGBKT7 vector as bait and potential interacting proteins into pGADT7 vector as prey. Then, bait and prey plasmids were co‐transformed into the yeast AH109 competent cells, which were cultured on SD‐Leu‐Trp‐His plates at a temperature 30 °C for 2–3 days with SD‐Leu‐Trp plates as controls.

### TEM

For TEM, samples were fixed in 2.5% glutaraldehyde (Solarbio, Beijing, China) overnight at 4 °C. After dehydrated in ethanol and embedded in epoxy resin, ultra‐thin slides were sectioned and stained with 0.3% lead citrate. Ultrastructure was visualized by transmission electron microscope (JEOL, Tokyo, Japan).

### In Vivo RNA Interference

The efficacies of customized *Csnk1g1* siRNAs (GACCGAACGUUUACUUUGATT, Genepharma, Shanghai, China) were evaluated by using the N2A cell line according to the manufacturer's instructions. To knock down *Csnk1g1* expression in vivo, we performed intratesticular injection of siRNA via the rete testis as previously described.^[^ ^48^, ^49^ ^]^ Three‐week‐old male mice were anesthetized with 1.25% tribromoethanol and had the testes exteriorized through a ≈1 cm midline abdominal incision. *Csnk1g1* siRNA mixed with 0.1% Fast Green was injected into seminiferous tubules via the rete testis, with the scramble siRNA (UUCUCCGAACGUGUCACGUTT) injected into the other testis. After injection, the testes were repositioned, the abdomen was sutured, and the mice were kept recovering and left undisturbed. The mice were sacrificed after 72 h for protein analysis or after 3 weeks for epididymal sperm morphology evaluation.

### Protein Extraction and Digestion

For proteomics and phosphoproteomics analysis, spermatids from four defined developmental stages were collected independently from testes of 8–10 mice in each of the four biological replicates. The collected cells were dissolved in lysis buffer (8 m Urea, 75 mm NaCl, 50 mm Tris, pH 8.2, 1% EDTA‐free protease inhibitor, 1 mm NaF, 1 mM β‐glycerophosphate, 1 mm sodium orthovanadate, 10 mm sodium pyrophosphate). After ultrasonic treatment, the cell lysate was centrifuged at 30 000 g for 60 min at 4 °C. The supernatant was collected, with the protein concentration measured by Bradford assay. The protein was trypsin digested with peptides desalted via an OASIS HLB Vac cartridge (Waters, Milford, MA).

### TMT Labeling, High pH Reversed‐Phase Chromatography and Phosphopeptide Enrichment

For TMT labeling, peptides from each sample were labeled with distinct TMT 16‐plex isobaric tags according to the manufacturer's instructions (Thermo Fisher, Waltham, MA), enabling simultaneous LC‐MS/MS analysis while preserving sample‐specific quantitative information. In detail, the TMT reagent was added to 100 µg of peptide at room temperature for 1 h in a 2:1 reagent‐to‐peptide ratio, and terminated by 5% hydroxylamine for another 15 min. The TMT‐labeled samples were combined at a 1:1 ratio, desalted, and dried using Speedvac concentrator (Thermo Fisher, Waltham, MA). The dried peptides were reconstituted in 5 mM ammonium formate and separated using an XBridge BEH130 C18 column facilitated by an Agilent 1260 series system. Fifteen fractions were collected over ≈34 min using a non‐adjacent pooling strategy. Each fraction was dried using a Speedvac concentrator to enrich phosphopeptides. The phosphopeptide enrichment was performed as previously described.^[^ ^9^ ^]^ In detail, peptides were suspended in 100 µL loading buffer (80% ACN, 3% trifluoroacetic acid) and mixed with Ti‐immobilized metal affinity chromatography (JKchemical, Beijing, China) in a 4:1 ratio, incubated for 20 min. The beads were washed with wash buffer I (50% ACN, 200 mm NaCl, 6% TFA) and II (30% ACN, 0.1% TFA), and phosphopeptides were eluted with 10% NH_4_OH. The eluents of phosphopeptides were dried and desalted for mass spectrometry analysis.

### LC‐MS/MS Analysis and Data Processing

For LC‐MS/MS analysis, peptides were resuspended in 0.1% formic acid and analyzed using the Orbitrap Fusion Lumos mass spectrometer and Easy‐nLC 1200 (Thermo Fisher, Waltham, MA). Solvent A was 0.1% formic acid, and solvent B was 80% ACN with 0.1% formic acid. Peptides were analyzed via a 75 µm×25 cm Acclaim PepMap RSLC C18 column using a 95‐min linear gradient followed by Orbitrap Fusion Lumos operated at 60 000 resolution with a 4 × 10^5^ ion AGC target and a 50 ms maximum injection time; MS2 scanning was done at 50,000 resolution.

Raw data were processed with MaxQuant using the Universal Protein Resource (UniProt) mouse database (Jan 2022) with Carbamylation (C) as a fixed modification, oxidation (M), acetylation (Protein N‐term), phosphorylation (Protein N‐term) as variable modifications, and complete trypsin cleavage with two missed sites. Only phosphorylation sites with localization probabilities over 0.75 were considered high confidence and subjected to downstream analysis. For quantification, the abundance of each protein was normalized by dividing it by its mean level across all samples. The relative abundance of each phosphorylation site was normalized in the same way and further calibrated by dividing it by the normalized abundance of its corresponding protein to account for protein‐level variation. If the protein abundance was zero in any sample, it was replaced with the lowest non‐zero value in the protein dataset to avoid invalid division. If the corresponding protein was not quantified, the normalized phosphorylation site level was used directly for downstream analysis.^[^ ^53^, ^54^ ^]^

### Bioinformatics Analysis

For temporal pattern analysis, significantly regulated proteins and phosphorylation sites were clustered using fuzzy c‐means clustering implemented in the Mfuzz package (version 2.6.1). Input data consisted of z‐score normalized abundance values that were averaged across experimental groups or time points. Clustering was performed with c = 5 clusters, and the fuzzifier parameter m was empirically estimated using the mestimate function.

WebLogo 3.7.11 was used to generate frequency plots of 21‐mer amino acids surrounding phosphorylation sites. Principal component analysis (PCA) was performed using the prcomp function in R. Gene ontology (GO) and Kyoto Encyclopedia of Genes and Genomes (KEGG) pathway enrichment analyses were performed utilizing the clusterProfiler 4.6.2 package (27) with an p‐value less than 0.05 as a cut‐off.

### Kinase Enrichment and Module Analysis

Kinase enrichment and module analysis are performed as previously described.^[^ ^53^, ^54^ ^]^ In brief, kinase enrichment analysis was performed to identify kinases significantly associated with phosphorylation modules derived from Mfuzz clustering. Kinase‐substrate relationships were annotated using GPS 5.0 with a medium confidence threshold. For each kinase and phosphorylation module (e.g., Module 1), a 2 × 2 contingency table was constructed comparing the number of phosphorylation sites regulated by the kinase within the module to those outside all significant modules (i.e., non‐significant sites not assigned to Modules 1–4).

Fisher's exact test was used on these counts to evaluate whether the kinase was significantly enriched in the module compared to the background. The odds ratio (OR) indicates the enrichment strength and direction, with an OR > 1 suggesting hyperrepresentation (activation) and an OR < 1 suggesting hyporepresentation (inhibition). Kinases with OR > 1.2 or OR < 1/1.2 and p‐value < 0.05 were considered significant for the given module.

### Western Blotting

Cells or tissues were lysed on ice in RIPA buffer (50 mm Tris–HCl pH 7.4, 150 mm NaCl, 1% NP‐40) containing protease and phosphatase inhibitors, then centrifuged at 16 000 g for 1 h at 4 °C. Protein concentration was determined by BCA assay, and 30 µg per lane was resolved on 6–12% SDS–PAGE (90 V, ≈1 h). Proteins were transferred to PVDF membranes (90 V, 2 h, 4 °C), blocked in 5% skimmed milk/TBST for 1 h, and incubated with primary antibodies overnight at 4 °C. After 4 × 10 min washes in TBST, membranes were incubated with HRP‐conjugated secondary antibodies for 2 h at room temperature, followed by 4 × 10 min TBST washes. Detection was performed using Chemistar™ High‐sig ECL Western‐blotting Substrate (Tanon, Shanghai, China) applied 1:1, then imaged on a ChemiDoc (Bio‐Rad, Hercules, CA) by exposure for 0.5–10 s. Phos^TM^‐Tag acrylamide was purchased from Wako (Osaka Prefecture, Japan). Phos^TM^‐Tag Western blotting was performed in gels containing 50 µM Phos‐tag and 7.5% acrylamide, and was followed according to the manufacturer's instructions. The densities of IFT88 phosphorylated proteoform relative to total IFT88 proteoform were determined by quantification with Fiji.^[^ ^50^ ^]^ Statistical analyses were performed by GraphPad Software. A two‐tailed Student's t‐test was used to compare two groups.

### Cell Culture and *Ttbk2^−/−^* Cell Line Construction

HEK293T and NIH3T3 cells (ATCC, Manassas, VA) were cultured in DMEM (ed with 10% Fetal Bovine Serum, 1% penicillin‐streptomycin solution) at 37 °C with 5% CO_2_. Cells were tested negative for mycoplasma contamination using a PCR Mycoplasma detection kit (TransDetect, Beijing, China) and authenticated via STR profiling (Biowing, Shanghai, China). The *Ttbk2* knockout (*Ttbk2* ^−/−^) cell line was generated using CRISPR‐Cas9 technology in NIH3T3 cells. Two sgRNAs (sgRNA1 sequence: TGGACATGCTCACCAGGGAG and sgRNA2 sequence: GTCAGCTCAGCAGCCAAAGC) were designed via Synthego and cloned into the pSpCas9 (BB)‐2A‐GFP (PX458) vector.^[^ ^55^ ^]^ The plasmids were transfected into NIH3T3 cells, and single cells were sorted using a BD FACSAria Fusion Flow Cytometer 48 h post‐transfection. Clonal cell lines were genotyped by Sanger sequencing to confirm successful *Ttbk2* knockout. This cell line was considered as a stable cell line, and was consistently used throughout the study.

### Cilia Induction

Cilia induction was conducted following a previously published protocol.^[^ ^56^ ^]^ In brief, cells were first transfected with the plasmid of interest and incubated for 24 h in complete medium containing serum. The culture medium was then replaced with serum‐free DMEM, and the cells were further incubated for 48 h to induce primary cilia formation. Following serum starvation, cells were subjected to immunofluorescence analysis. The biological replicates were conducted with cells at the same passage number.

### Statistical Analysis

For differential analysis of abundances of proteins or phosphorylation sites, one‐way ANOVA followed by Tukey's post hoc test was performed for analyses among groups, and Benjamini–Hochberg (BH) adjustment was used to control the false discovery rate. To ensure biological relevance, FC was calculated as the ratio of the maximum/minimum group mean abundance across steps of spermiogenesis. Proteins or phosphosites with adjusted P values (P.adjust) < 0.05 and FC > 2 were considered significantly regulated. For comparisons between individual groups, significance was further supported by pairwise post hoc tests with P < 0.05 and FC > 2.

For other experiments (e.g., Western blot, immunofluorescence, fertility tests, cilliogenesis assays), two‐tailed unpaired Student's t‐tests were applied for pairwise comparisons. Each experiment was independently performed at least three times. For Western blot quantification, the intensities of protein bands were measured by Fiji,^[^ ^50^ ^]^ the relative abundance of each protein was normalized to the intensity of a loading control (e.g., GAPDH or β‐actin), and the abundance of the phosphorylated proteoform was normalized to that of the total proteoform. All data were represented as mean ± SD, as annotated in the related figure legends. For statistical analysis, two‐tailed unpaired Student's *t*‐tests were performed by GraphPad software, one‐way ANOVA, and kinase enrichment and module analysis were performed by R software. Statistical significance was defined as *p* < 0.05. Exact p‐value and sample size (n) for each analysis were listed in Table S8 (Supporting Information) and the related figure legends.

### Code Availability

All custom scripts used for phosphoproteomic data processing, kinase‐substrate enrichment analysis, and figure generation are available at: https://github.com/XuejiangGuo/PhosphoproteomicsAnalysis.

## Conflict of Interest

The authors declare no conflict of interest.

## Author Contributions

T.Z., Y.Z., X.J., X.Z., B.W., and Y.C. contributed equally to this work. X.G., Y.L., H.Z., and T.Z. conceived the project. T.Z., Y.Z., X.J., and Y.C. performed animal experiments, spermatid purification, and sample preparation. X.Z.Z. and Y.G. conducted phosphopeptide enrichment and LC‐MS/MS analysis. B.W., X.Z.Z., and T.Z. performed bioinformatics and phosphoproteomic data analysis. Y.C., Y.C.Z., Y.W., Q.Z., Z.H., Y.Q., M.L., H.T., B.H., M.G., J.R., X.Z., X.Y.Z., X.C., H.L., Q.H., C.S., and Y.G. contributed to sample preparation, phenotype experiment, data interpretation, and technical support. X.G., Y.L., H.Z., T.Z., Y.Z., and X.J. analyzed the data, prepared the figures, and the manuscript with input from all authors. X.G., Y.L., and H.Z. supervised the project. All authors have read and approved the final manuscript.

## Supporting information



Supporting Information

Supporting Information

Supplemental Table 1

Supplemental Table 2

Supplemental Table 3

Supplemental Table 4

Supplemental Table 5

Supplemental Table 6

Supplemental Table 7

Supplemental Table 8

## Data Availability

The mass spectrometry proteomics data have been deposited in the ProteomeXchange Consortium via the PRIDE partner repository. The dataset “TMT‐based phosphoproteomics and proteomics analysis of spermatids” is available under the accession number PXD063123 (reviewer username: reviewer_pxd063123@ebi.ac.uk, password: rJWtXmbUm5nD). The dataset “TMT‐based phosphoproteomics analysis of Ttbk2sko mouse testis” is available under the accession number PXD063193 (reviewer username: reviewer_pxd063193@ebi.ac.uk, password: TPzZhxe5upmb). Annotated spectra can be visualized using MS‐Viewer (https://msviewer.ucsf.edu/prospector/cgi‐bin/msform.cgi?form=msviewer) upon request with corresponding search keys.
