# Supplementary material for: Stage‐Resolved Phosphoproteomic Landscape of Mouse Spermiogenesis Reveals Key Kinase Signaling in Sperm Morphogenesis
Source: Adv Sci (Weinh). 2025 Sep 3;12(44):e08538. doi: 10.1002/advs.202508538 (PMC12667522; doi:10.1002/advs.202508538)
Supplement: Supplementary file 1 — Supporting Information [file ADVS-12-e08538-s003.docx]

**Supplementary Figures**

**
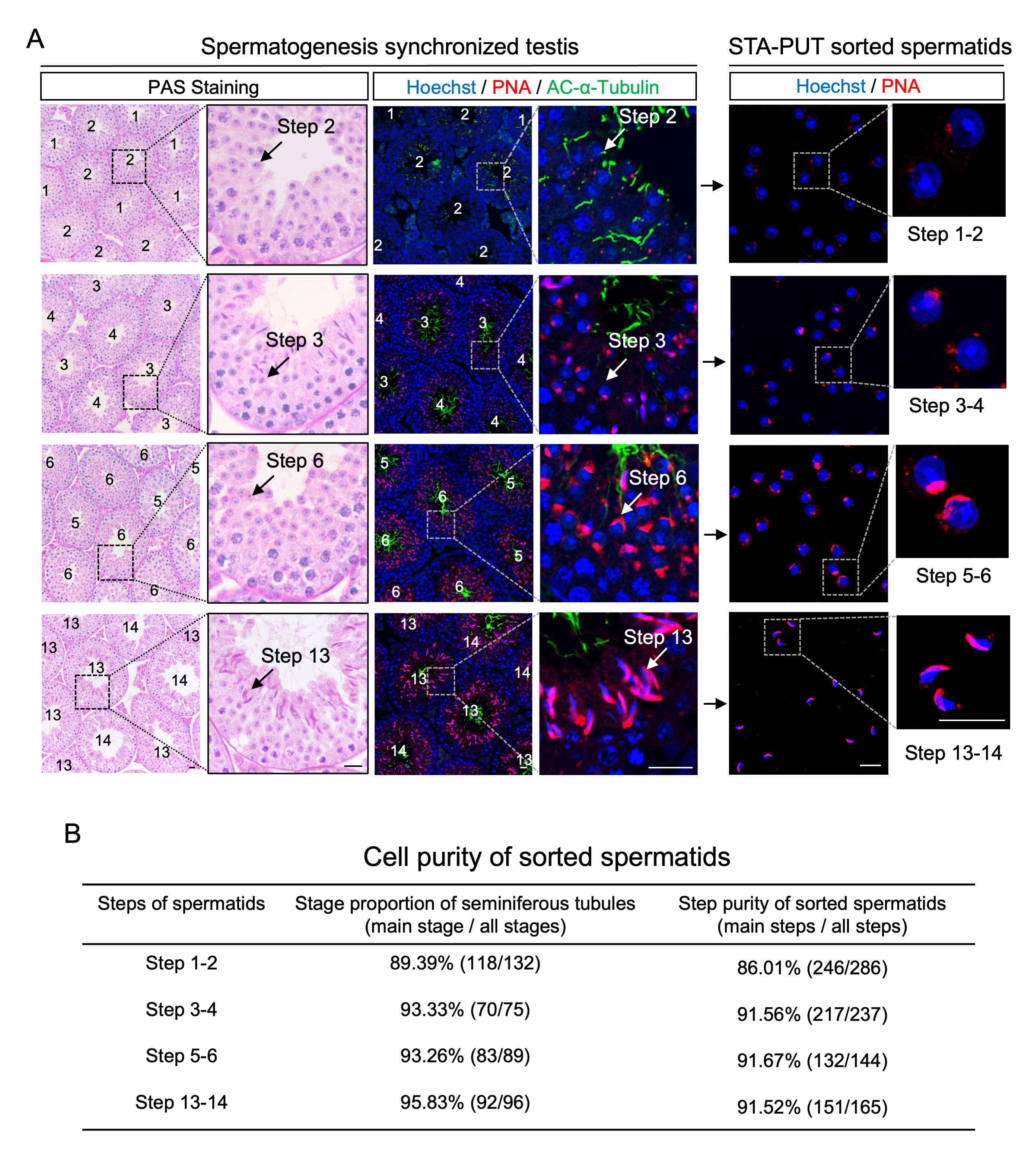
**

**Supplementary Figure 1. Purification of spermatids from spermatogenesis synchronized testes. A.** PAS staining and immunofluorescence of spermatogenesis synchronized testis sections and STA-PUT sorted spermatid at step 1-2, 3-4, 5-6, and 13-14. AC-α-Tubulin (green), PNA (red), and Hoechst (blue). (Scale bars = 20 µm) **B.** Statistics of stages of seminiferous tubules in spermatogenesis synchronized testis, and purity of purified spermatids (Stage purity of PAS staining).

**
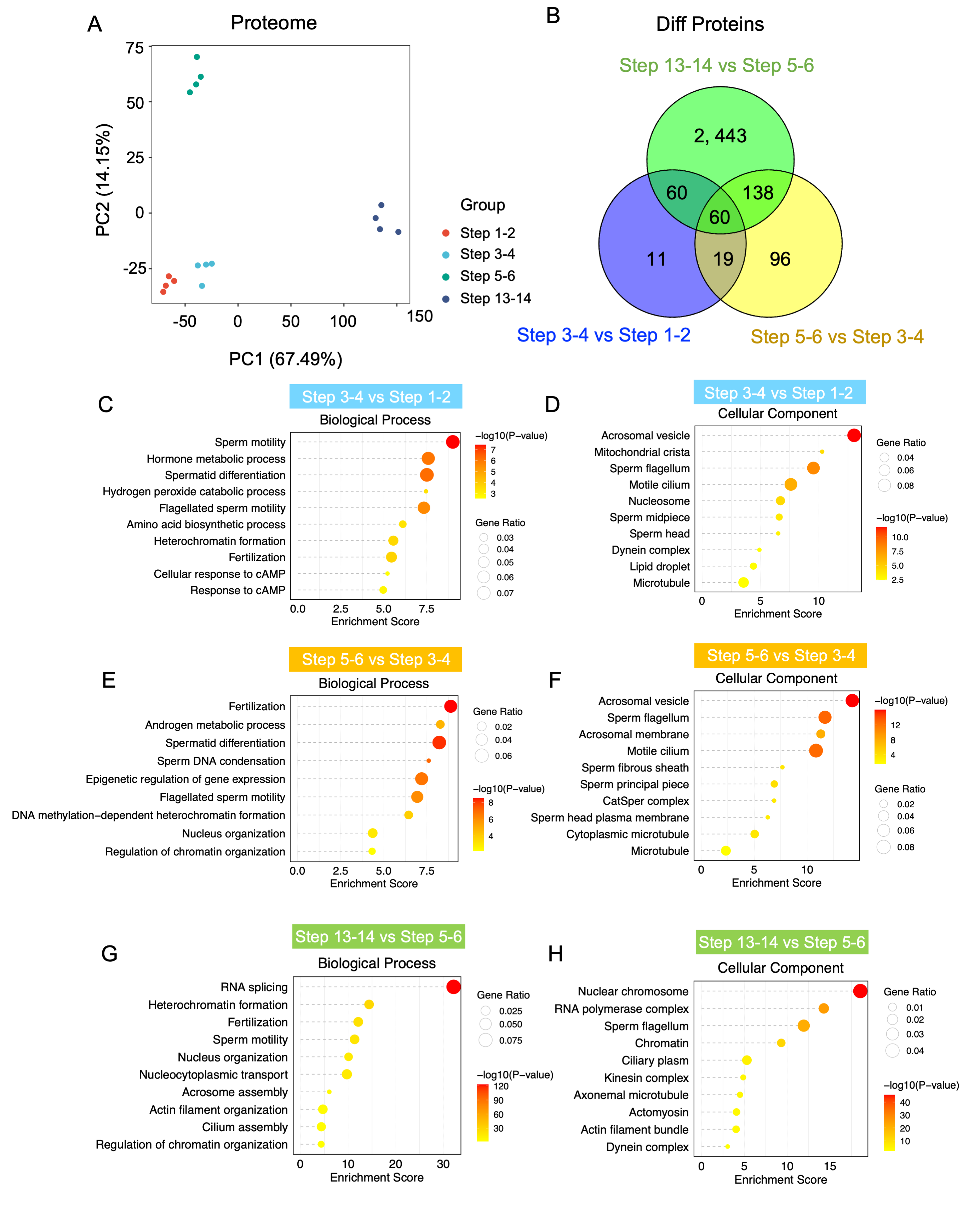
**

**Supplementary Figure 2**. **Proteomic profile of stepwise spermatid development by analyzing proteins with differential abundance (PDA) from adjacent steps.** **A.** Principal component analysis (PCA) of PDAs from different adjacent steps. **B.** Overlap of PDAs from different adjacent steps. **C–H.** Gene ontology (GO) analysis with Biological Process (**C**, **E**, **G**) and Cellular Component (**D**, **F**, **H**) enrichment for PDAs between adjacent stages: step 1–2 vs. step 3–4 (**C**, **D**), step 3–4 vs. step 5–6 (**E**, **F**), and step 5–6 vs. step 13–14 (**G**, **H**).

**
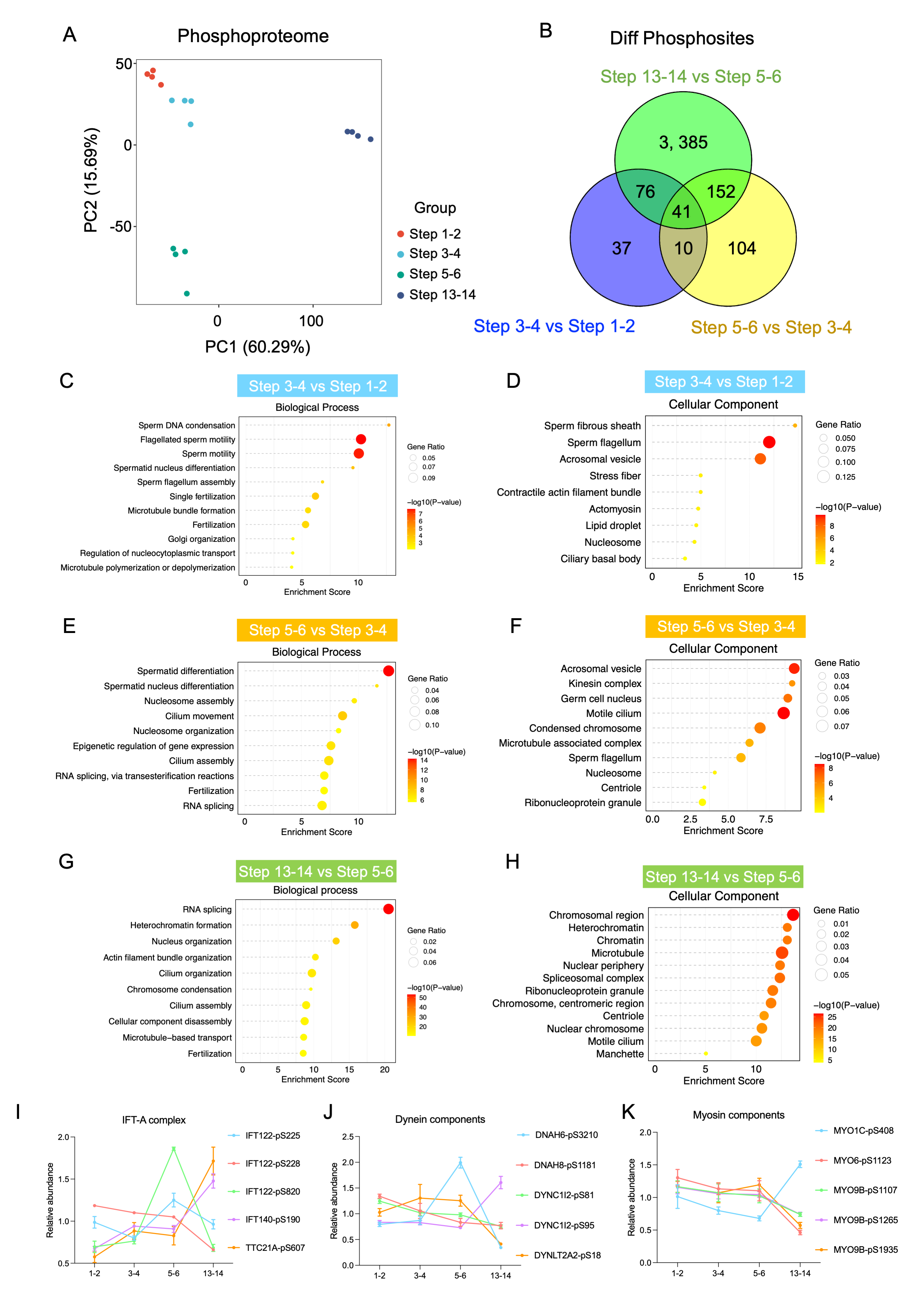
 Supplementary Figure 3**. **Phosphoproteomic profile of stepwise spermatid development by analyzing differential regulated phosphosites from adjacent steps.** **A.** Principal component analysis (PCA) of differential regulated phosphosites from different adjacent steps. **B.** Overlap of differential regulated phosphosites from different adjacent steps. **C–H**. Gene ontology (GO) analysis with Biological Process (**C**, **E**, **G**) and Cellular Component (**D**, **F**, **H**) enrichment for differential regulated phosphosites between adjacent stages: step 1–2 vs. step 3–4 (**C**, **D**), step 3–4 vs. step 5–6 (**E**, **F**), and step 5–6 vs. step 13–14 (**G**, **H**). **I-K**. Relative abundance changes of phosphorylation sites in IFT-A and Dynein proteins. Relative abundance (mean ± SD based on 4 biological replicates) changes of phosphorylation sites in IFT complex A (IFT-A) proteins (**I**), Dynein component protein (**J**), and Myosin components (**K**)across four developmental stages.


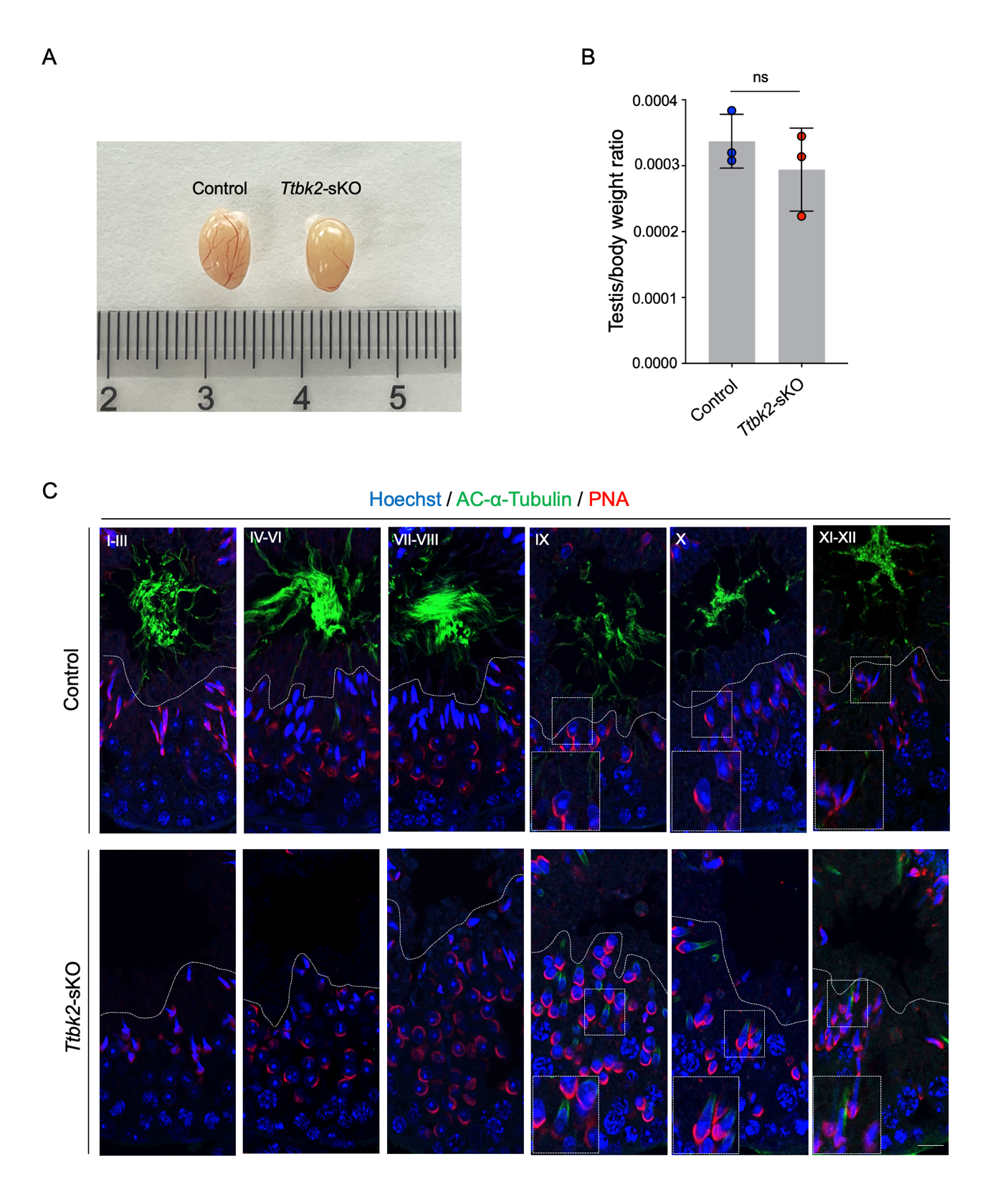


**Supplementary Figure 4. Testis and spermatid morphologies in *Ttbk2*-sKO mice**

**A.** The morphologies of 8-week *Ttbk2*-sKO and Control testes. **B.** Testis to body weight ratio of 8-week *Ttbk2*-sKO and Control mice (n = 3 for each sample, two-tailed Student’s t-test, data are presented as mean ± SD, p = 0.3765). ns, not significant. **C.** The immunofluorescence of AC-α-Tubulin (green) in seminiferous tubules at different stages with Hoechst (blue) and PNA (red). (Scale bar = 20 μm).

**
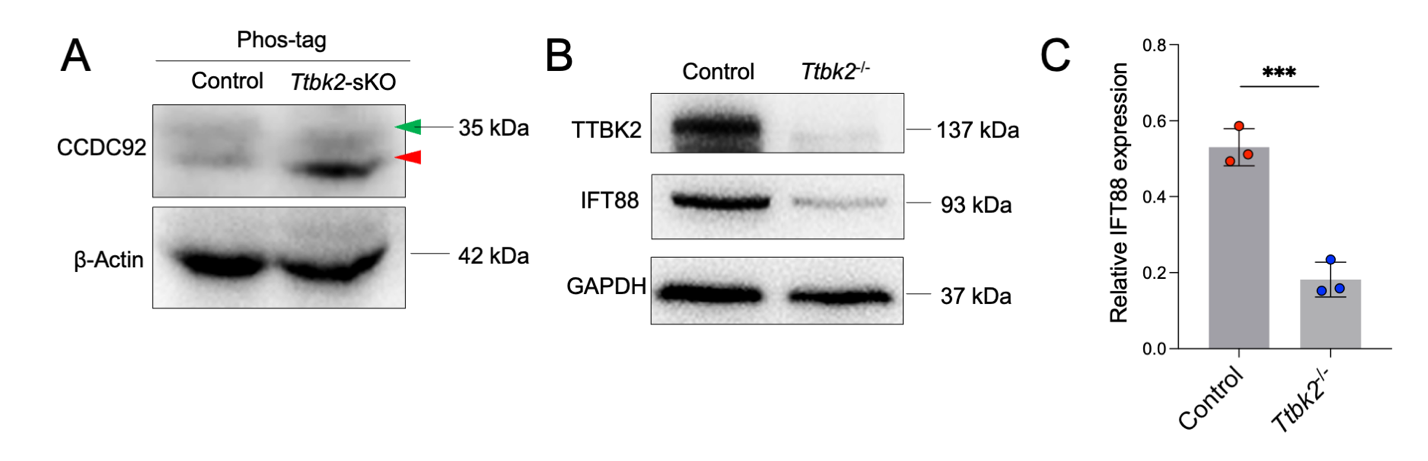
**

**Supplementary Figure 5.** **Positive control of phospho-shift in** ***Ttbk2*-sKO mice and expression of TTBK2 and IFT88 proteins in *Ttbk2*^-/-^ cell line.**

**A.** CCDC92 immunoblotting of *Ttbk2-*sKO and Control testicular lysate samples in Phos^TM^-Tag immunoblot**,** β-actin was used as the loading control. Green arrowhead, band of phosphorylated CCDC92 proteoform; Red arrowhead, band of non-phosphorylated CCDC92 proteoform.  **B-C.** Western blot (**B**) and quantification (**C**) of TTBK2, IFT88 and GAPDH proteins in *Ttbk2*^-/-^ cell line (n = 3, two-tailed Student’s t-test, data are presented as mean ± SD, p = 0.0008, ***, p < 0.001). The relative abundance of IFT88 (**C**) was normalized to the intensity of GAPDH.

**Supplementary Tables**

**Supplementary Table 1. Mouse spermatid proteome of different development stages.**

**Supplementary Table 2. The enriched BP (a) and CC (b) of spermatid of different developmental stages.**

**Supplementary Table 3. Phosphorylation sites identified in mouse spermatid phosphoproteome of different development stages.**

**Supplementary Table 4. The enriched KEGG pathway (c) and CC (b) in spermatid phosphoproteome of different developmental stages.**

**Supplementary Table 5. Kinases with enriched phosphorylated substrates in different quantitative phosphorylation modules.**

**Supplementary Table 6. Quantitative phosphoproteome (a), Class I phosphosites (b) and proteome (c) of *Ttbk2*-sKO and Control testes.**

**Supplementary Table 7. The enriched cellular CC (a) and BP (b) of in differential phosphoproteome between *Ttbk2*-sKO and Control testes.**

**Supplementary Table 8. Full list of exact p-values (a) and sample size (n) (b) for statistical analyses in this study.**
