# Supplementary material for: Stage‐Resolved Phosphoproteomic Landscape of Mouse Spermiogenesis Reveals Key Kinase Signaling in Sperm Morphogenesis
Source: Adv Sci (Weinh). 2025 Sep 3;12(44):e08538. doi: 10.1002/advs.202508538 (PMC12667522; doi:10.1002/advs.202508538)

Figure 4B-Source data

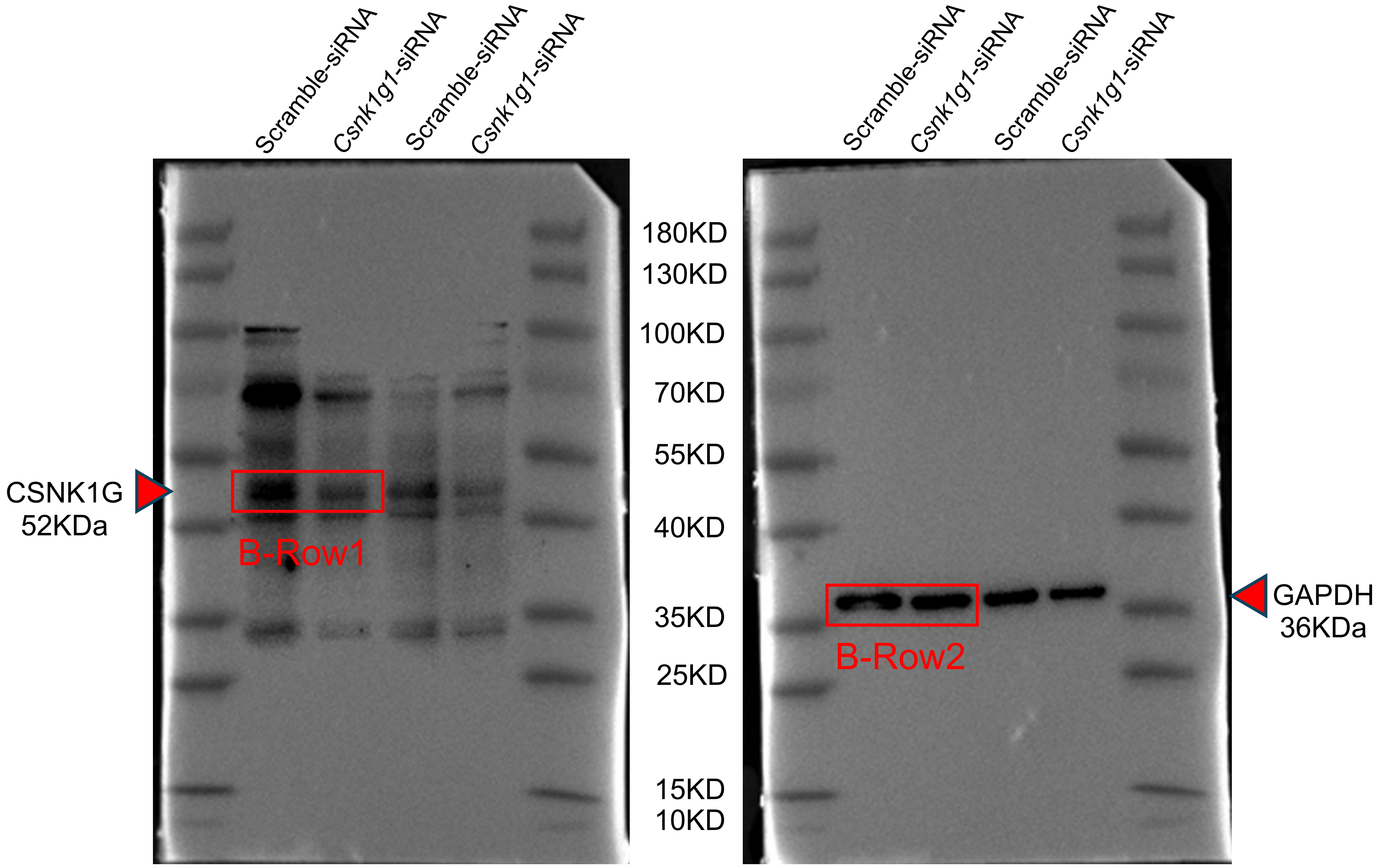

Figure 5C-Source data

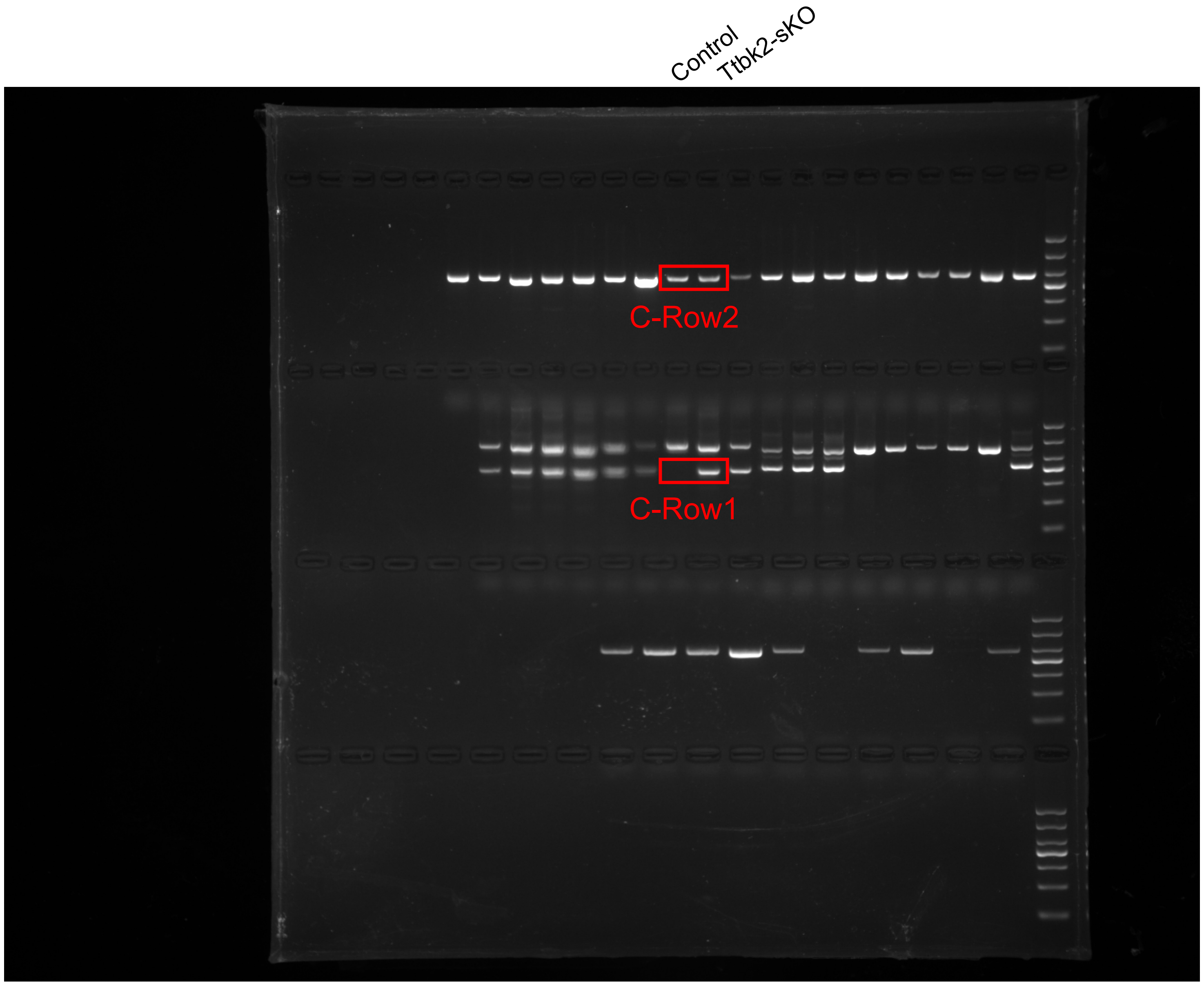

Ttbk2^flox/flox

Stra8-Cre

Figure 5D-Source data

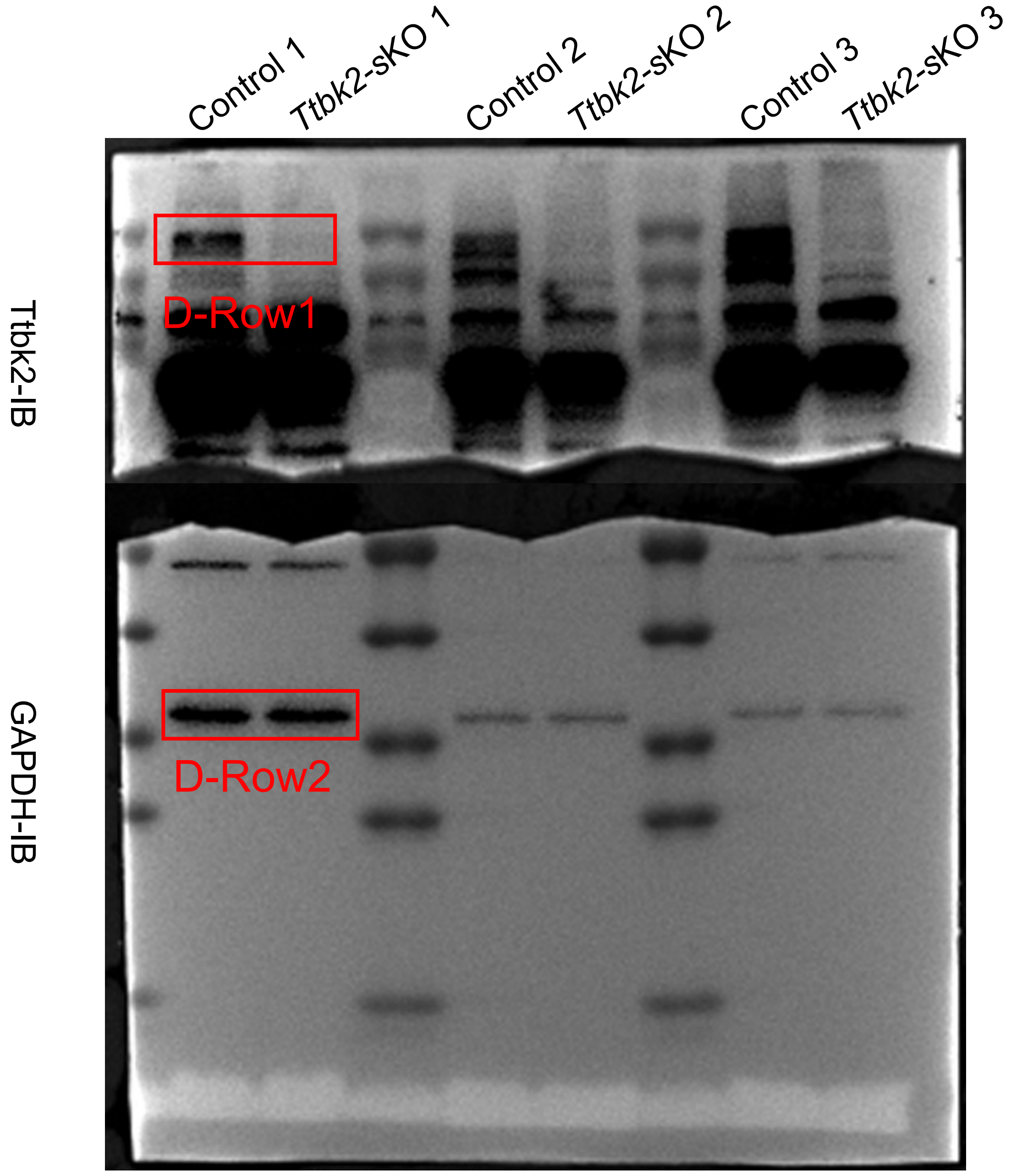

Figure7B-Source data

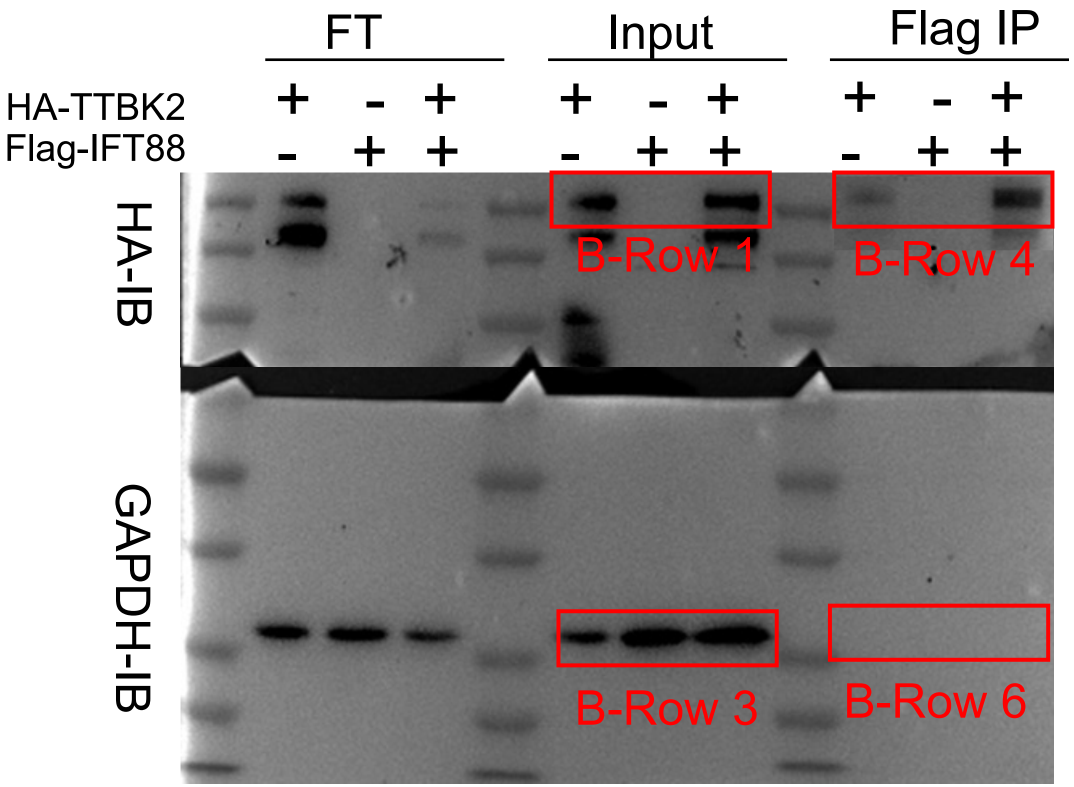

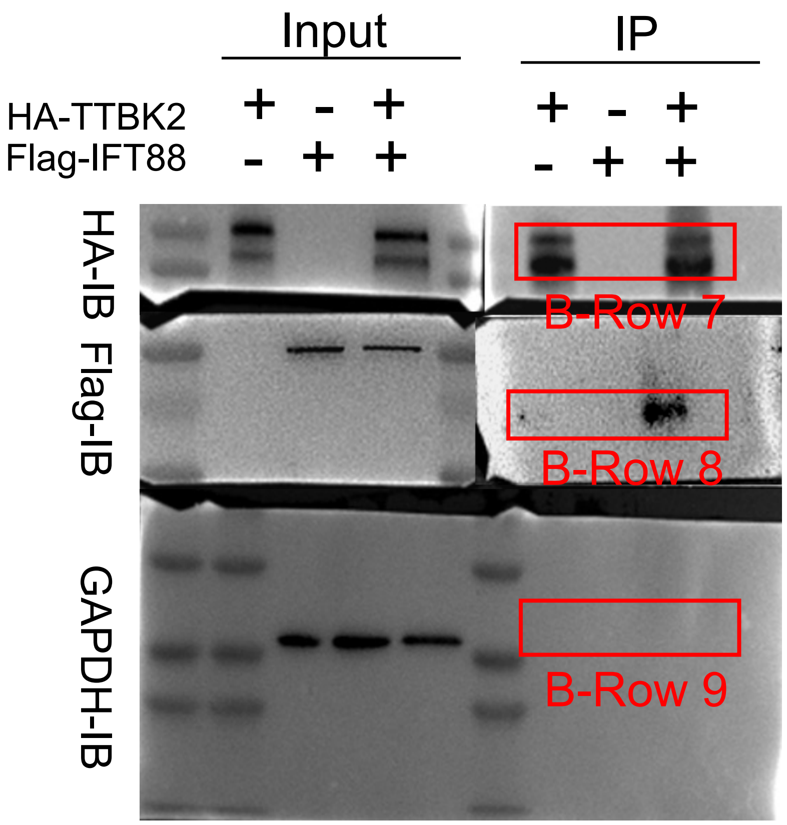

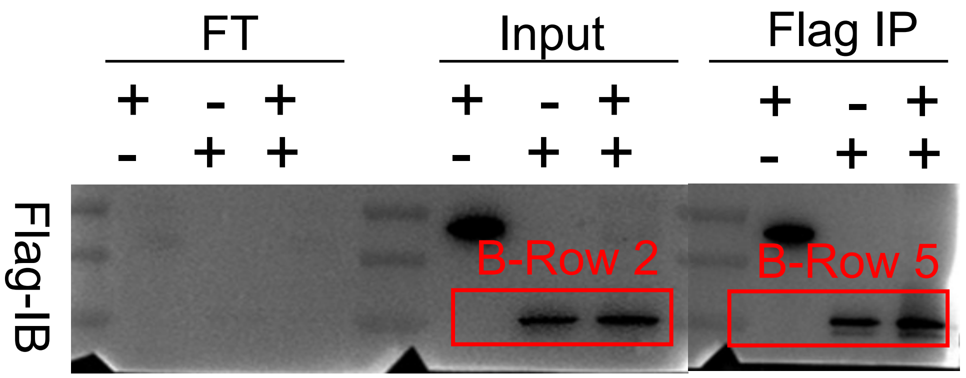

Figure7C-Source data

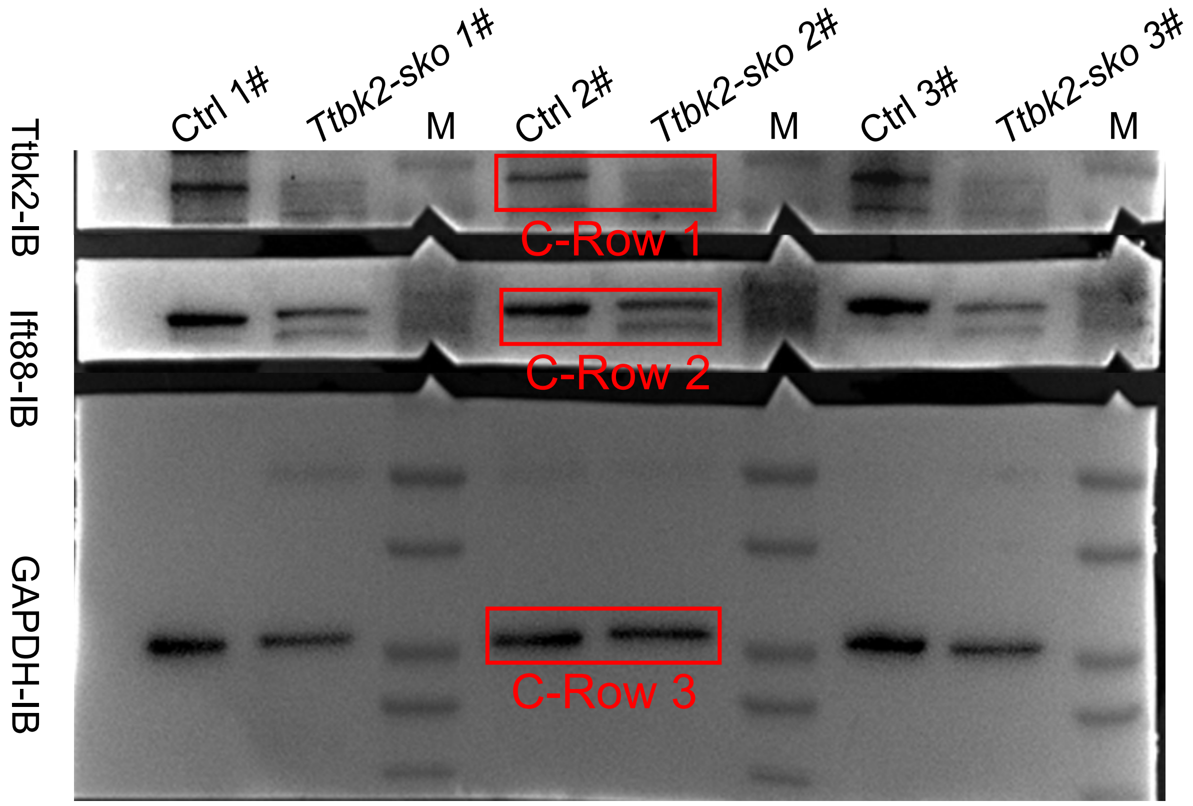

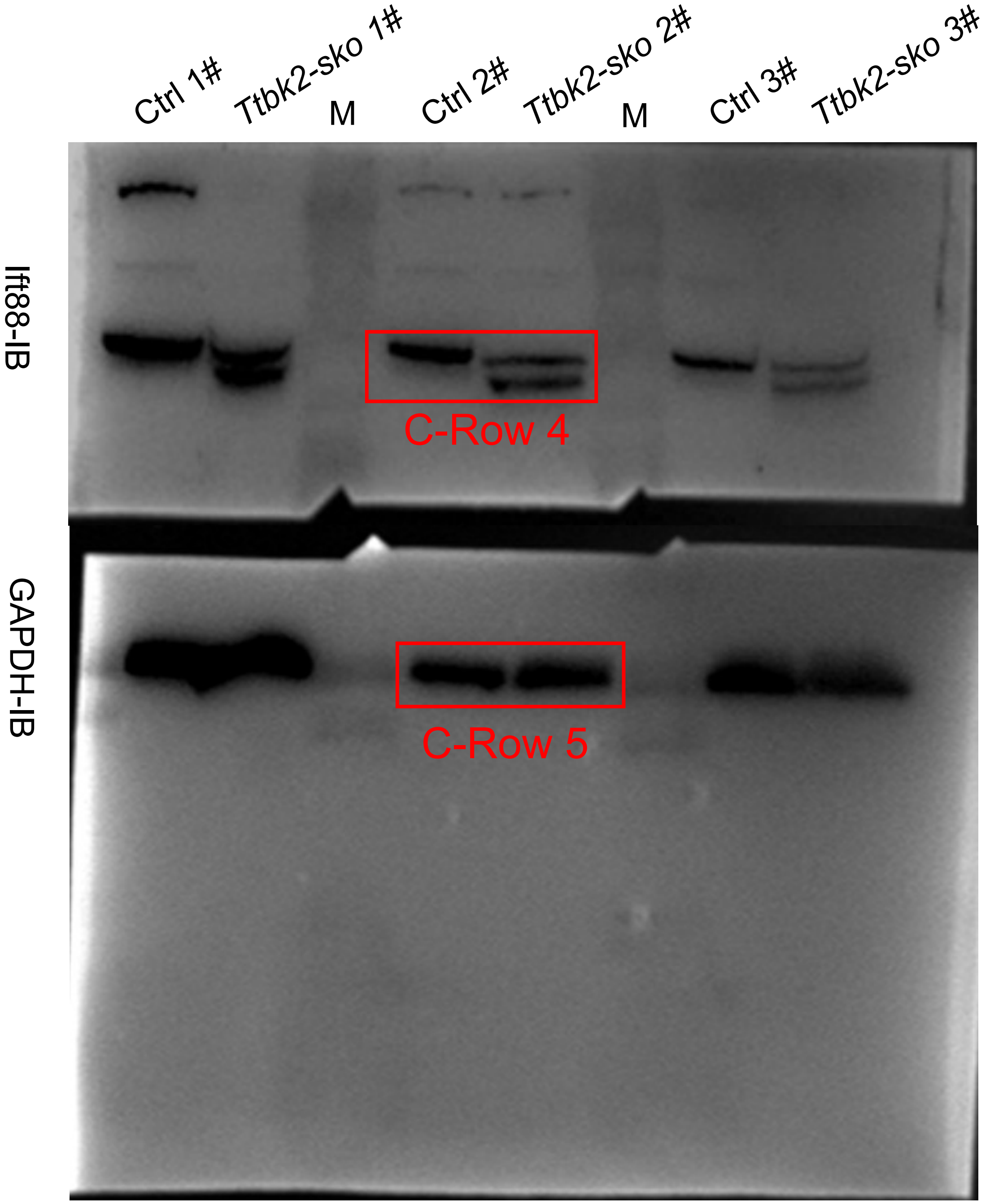

Figure7E-Source data

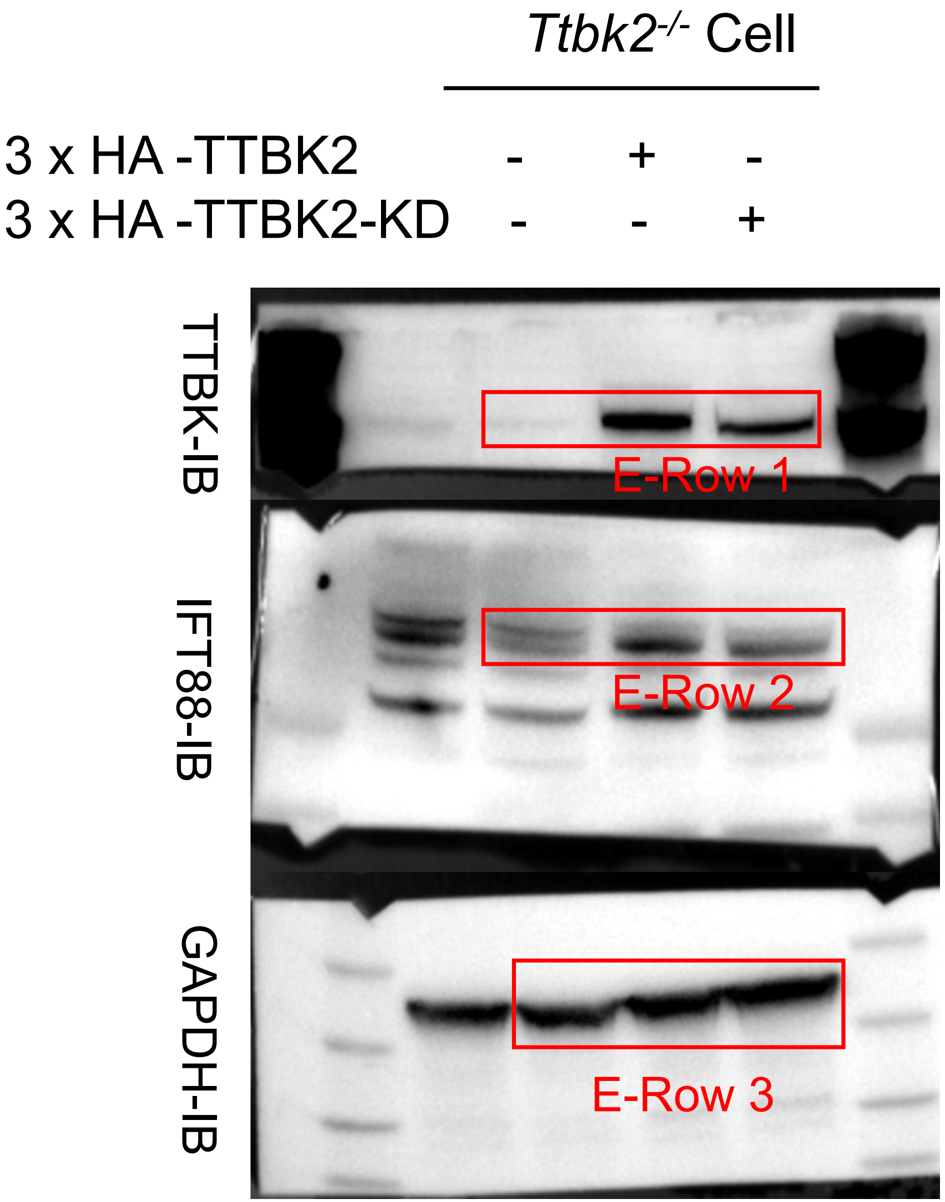

Supplementary Figure 5A-Source data

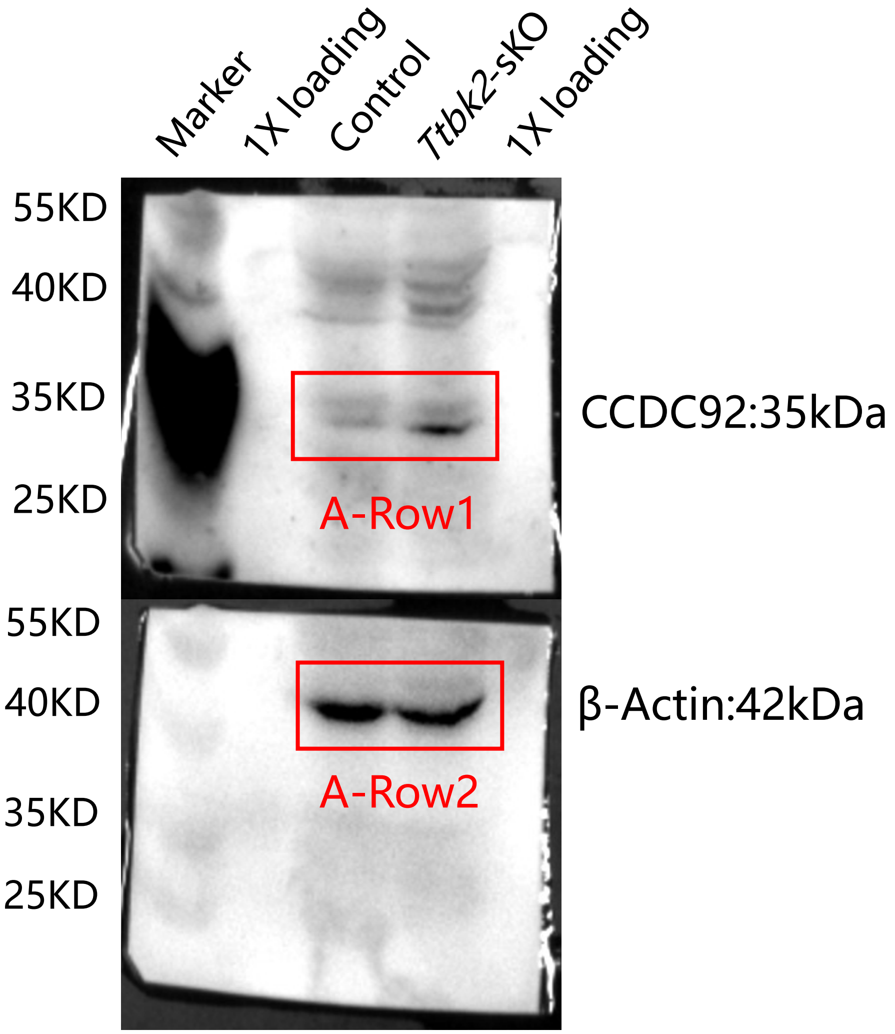

Supplementary Figure 5B-Source data

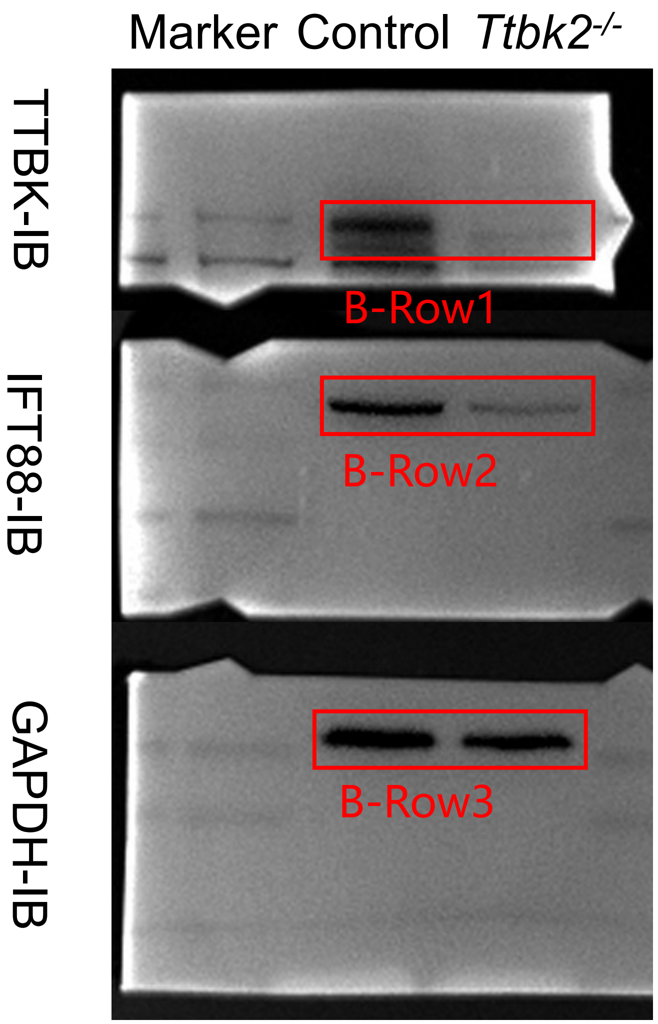

Supplement: Supplementary file 2 — Supporting Information [file ADVS-12-e08538-s008.zip › advs71690-sup-0001-data.pdf]
